# Inhibition of SOD1 trimerization is a novel drug target for ALS disease

**DOI:** 10.1186/s40035-025-00483-8

**Published:** 2025-05-12

**Authors:** Tae-Gyun Woo, Jin Han, Yuju Kim, Young jun Hwang, Mua Lee, So-mi Kang, Soyoung Park, Yeongseon Ji, Yeon-Ho Chung, Songyoung Baek, Eunbyeol Shin, Minju-Kim, Hyewon Jang, Yun-Jeong Shin, Yonghoon Kwon, Bae-Hoon Kim, Bum-Joon Park

**Affiliations:** 1 Institute of Rare Genetic Disease, PRG S&Tech Co., LTD, Busan, 46274 Republic of Korea; 2 Department of Molecular Biology, College of Natural Science, Pusan National University, Busan, 46241 Republic of Korea; 3 Department of Agricultural Biotechnology, Seoul National University, Seoul, 08826 Republic of Korea

**Keywords:** Amyotrophic lateral sclerosis disease, SOD1 aggregation, SOD1 trimer, Neurodegeneration, Non-clinical study

## Abstract

**Background:**

Amyotrophic lateral sclerosis (ALS) is a progressive neurodegenerative disease that begins with motor neuron death in the spinal cord and cerebral cortex, ultimately resulting in death from respiratory distress (breathing failure). About 90% of ALS cases are sporadic, and 10% of ALS cases are of the inherited type with a genetic cause. About 150 different gene mutations have been reported so far. *SOD1* is a well-identified gene associated with ALS. Indeed, SOD1 aggregation has been reported in ALS patients, but the mechanism of SOD1 aggregation remains unclear. Our previous work showed that inhibiting SOD1 aggregation with a hit compound (PRG-A-01) could reduce the SOD1-induced cytotoxicity and extend the lifespan of ALS mouse model (SOD1^G93A−Tg^). However, the low bioavailability and rapid degradation of the compound in vivo necessitates the development of a more effective candidate. We generated different derivatives and finally obtained the most potential drug candidate, PRG-A-04.

**Methods:**

Neuronal cell lines were transfected with the mutant SOD1 expression vector and incubated with PRG-A-04. SOD1 aggregation was examined by SOD1 oligomerization assay, immunofluorescence and dot blot assay. The interaction between GST-conjugated SOD1 recombinant proteins and PRG-A-04 was identified using LC–MS/MS and GST pull-down assay. To check the in vivo therapeutic effect of PRG-A-04, SOD1^G93A−Tg^ mice were injected with PRG-A-04; then behavioral test, histological analysis and microarray were performed.

**Results:**

PRG-A-04 demonstrated favorable pharmacokinetics including high bioavailability and significant blood–brain barrier penetration. Indeed, oral administration of PRG-A-04 in ALS mouse model inhibited the aggregation of SOD1 in the spinal cord, protected against neuronal loss, and extended the lifespan of ALS mice by up to 3 weeks. In vitro, PRG-A-04 selectively bound to the mutant form of SOD1, but not the wild type, and efficiently inhibited the aggregation caused by SOD1-G147P (a SOD1 trimer stabilizer).

**Conclusions:**

Our findings underscore the potential of targeting trimeric SOD1 in ALS treatment, positioning PRG-A-04 as a strong drug candidate for both familial and sporadic ALS.

**Supplementary Information:**

The online version contains supplementary material available at 10.1186/s40035-025-00483-8.

## Introduction

ALS is a progressive neurodegenerative disease characterized by muscle paralysis and stiffness, loss of motor function and respiratory distress, ultimately leading to death within 2–5 years after onset [1–4]. ALS progresses very rapidly, and to date, there is no adequate treatment to stop or reverse the progression of the disease, with a lack of clear understanding of the pathogenesis [5–7]. ALS is a complex disease that develops from a variety of causes and exhibits significant clinical heterogeneity in, e.g., the site and age of disease onset and the disease progression, making it difficult to diagnose [1, 8]. While many clinical trials have been conducted to develop a cure for ALS, the FDA has approved only four drugs: Riluzole, Edaravone, Relyvrio and Tofersen [9–17]. Relivrio was voluntarily withdrawn from the market after it failed to demonstrate efficacy in a phase 3 trial [15, 16, 18]. Current treatments for ALS focus on symptom management, with approved drugs neither curing the disease nor slowing its progression, but only acting for symptomatic relief. As such, ALS is a disease with a very high unmet medical need for new and effective treatments [5–7].

Abnormal protein aggregation can lead to several types of human disease, including neurodegenerative diseases (NDDs), such as extracellular Aβ plaques and intracellular tau tangles in Alzheimer’s disease (AD) or intracellular Lewy bodies in Parkinson’s disease [19–22]. Although 90% of ALS are sporadic without genetic mutation, protein aggregation would be also a cause of this disease [23, 24]. It is still controversial whether SOD1 aggregation is actually found in all ALS patients [25]. Furthermore, ALS is a complex disease with multiple causes and histopathological heterogeneity, making it difficult to classify most patients with *SOD1* mutations as having clinically typical ALS. It has been reported that ALS patients carrying *SOD1* variants have typical ALS clinical features, such as spinal onset (limb weakness) and bulbar onset. However, the clinical phenotype, such as disease progression, age and site of onset, varies between carriers of different *SOD1* variants [26]. Nevertheless, SOD1 aggregation has been detected in ALS patients without genetic mutation in the *SOD1* gene [27–31]. In this regard, we previously reported that under stress conditions such as Ca^2+^ or Zn^2+^ ion chelator treatment, wild-type (WT)-SOD1 could easily form aggregates and promote neuronal cell death [32]. In addition, mislocalized TDP-43 by its overexpression also promotes WT-SOD1 aggregation [28–31, 33], suggesting that SOD1 aggregation may be a common culprit factor in both sporadic ALS (sALS) and familial ALS (fALS). However, the mechanisms of SOD1 misfolding/aggregation remain unknown, despite several research explorations [23, 24, 34–38].

The SOD1 trimer, rather than the large complex of SOD1, is critical for SOD1 aggregation in the early stages [39, 40]. The trimer-stabilizing mutant G147P, but not the trimer-destabilizing mutants N53I and D101I, induces neuronal cell death [39–42]. Therefore, the pathological SOD1 protein aggregation reported in ALS patients (A4V, G85R, G93A, etc.) may be induced by cytotoxic SOD1 trimeric intermediates [43], necessitating research on the trimerization of SOD1 for understanding the progression of ALS disease.

We have previously reported that the novel chemical PRG-A-01 acts as an inhibitor of SOD1 aggregation. Treatment with PRG-A-01 could extend the lifespan of SOD1^G93A−Tg^ mice by about 10 days [32]. However, this chemical is not suitable for drug development due to a very short half-life in the in vivo system and poor pharmacokinetics. Therefore, it is necessary to optimize PRG-A-01 for the improvement of its pharmacokinetics.

In this study, we developed the optimized chemical PRG-A-04 as an SOD1 trimer inhibitor, and tested the effect of its oral administration on the progression of ALS disease and the lifespan of SOD1^G93A−Tg^ ALS mouse model.

## Materials and methods

### Transgenic mice

The experiments were performed in the Association for Assessment and Accerditation of Laboratory Animal Care certified facility, in compliance with animal policies approved by Pusan National University. B6SJL-Tg (SOD1^G93A^) mice were obtained from Jackson Laboratory (Sacramento, CA, Stock No: 002726). All mice were maintained under temperature- and light-controlled conditions (20–23 °C, 12 h light/dark cycle) with free access to food and water.

### Drug treatment in vivo and histological analysis

To evaluate the therapeutic effect of PRG-A-04, SOD1^G93A−Tg^ mice received intraperitoneal injection of vehicle (DMSO, *n* = 6) or PRG-A-04 (20 mg/kg, *n* = 4; 50 mg/kg, *n* = 6) twice per week from 11 weeks for 6–8 weeks. WT mice (*n* = 5) served as negative littermates of the SOD1^G93A−Tg^ mice. WT mice were treated in the same conditions.

For histology analysis, SOD1^G93A−Tg^ mice received intraperitoneal injections starting from 11 weeks and sacrificed at 17 weeks (vehicle, *n* = 3; PRG-A-04 20 mg/kg, *n* = 2, 50 mg/kg, *n* = 2) and 19 weeks (vehicle, *n* = 2; PRG-A-04 50 mg/kg, *n* = 2). WT male mice (*n* = 5) were sacrificed at 17 weeks. Mice were sacrificed by i.p. (intraperitoneal) injection of avertin at a volume of 150–200 μL. The spinal cord was obtained and fixed in 4% paraformaldehyde for 48 h and embedded in paraffin blocks. The embedded tissues (cervical region of the spinal cord) were sectioned at 5 µm by Accu-Cut ® SRMTM 200 Rotary Microtome (Sakura Finetek, Torrance, CA) and transferred onto adhesive-coated slides (Marienfeld Laboratory Glassware, Lauda-Königshofen, Germany). After deparaffin and rehydration, the slides were stained with hematoxylin and eosin (H&E) to detect spinal cord neurons. In addition, immunohistochemistry (IHC) with antibodies for neuronal markers MAP2 (ab32454, Abcam, Cambridge, UK) and NeuN (ab177487, Abcam) as well as for SOD1 (GTX100554, Genetex, Irvine, CA) was performed. The intensity of MAP2 and NeuN was calculated with the Image J software (version 1.54p, National Institute of Mental Health, MD). For counting intensity, IHC images were divided using the “color deconvolution” function in Image J software and the DAB signal was quantified.

To assess the efficacy of PRG-A-04 (amorphous solid dispersions, ASD) administered orally, vehicle (1% HPMC [hydroxy propyl methyl cellulose] E5/ 0.5% PVP [polyvinylpyrrolidone] K30/ 0.2% SLS [sodium lauryl sulfate] in water, *n* = 6) or PRG-A-04 (15 mg/kg *n* = 4, 25 mg/kg *n* = 5)] was administered (p.o. injection; Per os) twice per week from 9 weeks until the end-stage phenotype. To monitor the combined therapeutic effect of feeding and i.p. injection of PRG-A-04, SOD1^G93A−Tg^ ALS mice were fed daily with pre-mixed drug-diet pellets starting at 7 weeks of age, and i.p. injections were started at 11 weeks of age twice weekly to determine the longevity (*n* = 3 for both vehicle and PRG-A-04 groups). The composition of the pre-mixed drug-diet pellet is shown in Figure S7b.

### Motor performance assessment

SOD1^G93A−Tg^ mice were treated with PRG-A-04 by i.p. or combined therapy. After training for 2 weeks from the age of 9 weeks, the rotarod test was started from 11 weeks and the latency to fall was recorded with a rotarod machine. For mice with p.o. injection, the mice were trained for 2 weeks from the age of 7 weeks, and the rotarod test was performed from 9 weeks. Age-matched WT mice were tested as positive control. The rotation speed was gradually increased (5–50 rpm) for up to 5 min until the mouse fell. Mouse movement was recorded. The recorded video file was further analyzed using the EthoVision XT 15 software (Noldus, Wageningen, Netherlands).

### Cell culture and reagents

HEK293 cells were obtained from the American Type Culture Collection (ATCC, Manassas, VA) and maintained in DMEM medium (10% fetal bovine serum and 1% penicillin–streptomycin) at 37 °C and 5% CO_2_. SK-N-SH and SH-SY5Y cells were purchased from the Korean Cell Line Bank (KCLB, Seoul, South Korea). SK-N-SH cells were maintained in MEM medium (10% fetal bovine serum, 1% antibiotics, 25 mmol/L HEPES and 300 mg/L* L*-Glu). SH-SY5Y cells were cultured in a 1:1 mix of Advanced DMEM/F12 medium (Cat no 126324010, Gibco, ThermoFisher, MA) and DMEM/F12 medium (Cat no 11320033, Gibco, ThermoFisher) containing 10% fetal bovine serum and 1% penicillin–streptomycin. Human fibroblast cells (from a 22-year-old female) were obtained from the Coriell Cell Repositories (Camden, NJ) and maintained in EMEM containing 15% FBS, 2 mmol/L glutamine and 26 mmol/L HEPES without antibiotics. Thapsigargin (endoplasmic reticulum Ca^2+^ depletor; T9033, CAS 67526–95-8) was purchased from Calbiochem (Darmstadt, Germany). HaloTag-Fluorescent ligand (diAcFAM, G8272) was obtained from Promega (Madison, WI). Dynasore (dynamin inhibitor; 324410, CAS 304448–55-3) and Iodoacetamide (I1149, CAS 144–48-9) were purchased from Sigma Aldrich (St. Louis, MO).

### Recombinant proteins

The following recombinant protein expression cassettes were subcloned into the pCold GST vector (Takara Bio, Ellsworth, MI): GST, SOD1-WT-Halo, G93A-Halo, A4V, G37R, G85R, G93A, N53I, D101I, G147P. Recombinant proteins were expressed in the *Escherichia coli* (E.*coli*) strain BL21 (DE3) as GST-fusion proteins. The proteins were purified by glutathione-affinity chromatography. Halo recombinant proteins (WT and G93A) were cleaved with HRV 3C Protease to eliminate His-GST. The soluble recombinant proteins were purified using glutathione agarose (Thermo Fisher) according to the manufacturer’s instructions.

For detecting the oligomerization assay promoted by mutant-type (MT)-SOD1 recombinant proteins in vitro, GST-SOD1 recombinant protein (4 μg/mL) was incubated with PRG-A-04 (10 μmol/L) for 30 min at room temperature in PBS and the samples were mixed with non-denaturing sample buffer. Those were separated in SDS-PAGE gel without boiling.

### Western blot analysis

For SDS-PAGE, proteins were extracted from cells with the RIPA buffer (50 mmol/L Tris–Cl, pH 7.5, 150 mmol/L NaCl, 1% NP-40, 0.1% SDS and 10% sodium deoxycholate). To detect the oligomer status, the cells were harvested with the TNNI buffer (50 mmol/L Tris–Cl, pH 7.5, 150 mmol/L NaCl, 0.3% NP-40, 100 mmol/L iodoacetamide, protease inhibitor cocktails) and reacted with 0.04% glutaraldehyde (Glu) for 30 min, and then SDS-PAGE was performed using the pan-SOD1 antibody. Samples were separated via SDS-PAGE and transferred to PVDF membrane. Blotted membranes were blocked by 3% skim milk containing TBST buffer for 1 h and incubated with specific antibodies. The following primary antibodies were used: pan-SOD1 (GTX100554) from Genetex (Irvine, CA); misfolded SOD1-specific antibody (B8H10) from MediMabs (Montreal, Canada); Actin (sc-1616), GST (sc-138), and GFP (Green fluorescent protein; sc-8036) antibodies from Santa Cruz Biotechnology (Santa Cruz, CA); TDP-43 antibody (10782–2-AP) from Proteintech (Rosemont, IL); anti-FLAG (Sigma; F3165) from Sigma-Aldrich (St. Louis, MO). HRP-conjugated goat anti-mouse, goat anti-rabbit and mouse anti-goat antibodies (Pierce, Thermo Fisher Scientific, Inc., Rockford, IL) were used as secondary antibodies. The blots were detected using an ECL assay (Advansta) and images were acquired using the AMERSHAM ImageQuant 800 (Cytiva) system and an X-Ray film detector.

To isolate insoluble SOD1 from spinal cord of SOD1^G93A−Tg^ mice, tissue lysates were resolved with TNNS lysis buffer (50 mmol/L Tris–Cl, pH 7.5, 150 mmol/L NaCl, 0.3% NP-40, 0.5% SDS, Protease inhibitor cocktails) and sonicated. After sonication, solubilized extracts were centrifuged at 14,000 rpm for 30 min, then pellet (insoluble) and supernatant (soluble) were separated. To detect oligomeric status of SOD1, solubilized tissue extracts by sonication were reacted with iodoacetamide (100 mmol/L) for 1 h and then incubated with 0.04% glutaraldehyde for 1 h. The samples underwent SDS-PAGE. Actin was used as a loading control.

### Dot blot analysis

To detect misfolded SOD1 expression, cells transfected with SOD1 vectors were treated with PRG-A-04 for 24 h. After incubation, cells were lysed with detergent-free NN buffer (50 mmol/L Tris–Cl, pH 7.5, 150 mmol/L NaCl, 0.3% NP-40), and then cell lysates were immobilized on nitrocellulose membrane using the Bio-Dot SF Microfiltration apparatus (Bio-Rad Laboratories, Hercules, CA). The membrane was washed with TBS and blocked by 5% BSA to remove background for 1 h. After blocking, the membrane was incubated with antibody for misfolded SOD1 (1:2000) or actin (1:10,000 in 1% skim milk containing TBST) for 30 min, then reacted with secondary antibody (goat anti-mouse IgG-horseradish peroxidase, 1:50,000 in 1% skim milk blocking buffer) for 30 min. Proteins were detected with ECL and X-ray film exposure. Actin was used as the loading control.

### LC–MS/MS analysis

To prepare a calibration curve within the PRG-A-04 concentration range of 1–50 ppb, standard solutions (1, 5, 10, 25, 50 ppb) were prepared by dilution with 90% methanol. GST, GST-SOD1 WT, and GST–SOD1-G93A recombinant proteins bound to GST agarose were incubated overnight with PRG-A-04 (20 μmol/L) in cold chamber (4 °C). The samples were washed three times to remove unbound PRG-A-04 from the proteins. The washed sample pellets were incubated with 90% methanol to elute the protein-bound PRG-A-04. The elutes from each sample were analyzed by LC/MS as follows. Both standard samples and control samples underwent the same sample processing procedures as the test samples. The LC–MS/MS analysis was performed on a Waters UPLC Acquity I-class plus system coupled to a Qtrap 6500 plus system (Sciex, Framingham, MA). An Aeris 3.6 μm 100 × 2.1 mm column (Phenomenex, Torrance, CA) was used for separation. The mobile phase consisted of solvent A (water containing 0.1% formic acid) and solvent B (acetonitrile containing 0.1% formic acid). Gradient elution was achieved as follows: 0–3.70 min, 90% A, 10% B; 0.37–6.0 min, 55% A, 45% B; 6.0–7.0 min, 5% A, 95% B; 7.0–10.0 min, 90% A, 10% B. The flow rate was 0.4 mL/min. The column temperature was 40 °C, and the sample injection volume was 10 µL. After each injection, the autosampler syringe was washed with methanol. The Qtrap 6500 plus system was operated in the positive ionization mode. The optimal MS conditions were as follows: ion spray voltage of 5500 V, temperature of 500 °C, curtain gas of 25.0 psi, ion source gas 1 of 50 psi, and ion source gas 2 of 50 psi. The MS system was controlled by the Analyst 1.6.1 software.

### Immunofluorescence staining

Cells on coverslips were washed with PBS, fixed with 4% PFA for 30 min at room temperature and then permeabilized in 0.1% Triton X-100/PBS for 10 min. After incubation in blocking solution (goat serum diluted 1:200 in PBS) for 1 h, cells were incubated with primary antibodies in blocking buffer for overnight at 4 °C. Finally, the cells were incubated with FITC- or Rhodamine-conjugated secondary antibody at 4 °C for 4 h. The nucleus was stained with 4, 6-diamidino-2-phenylindole and the endoplasmic reticulum (ER) was stained using the ER-Tracker Red dye for 10 min. Cells were washed three times with PBS, then the coverslips were mounted with the mounting solution (H-5501; Vector Laboratories, Burlingame, CA) and observed by fluorescence microscopy using Zeiss Apotome 2 (Software; Axiovision 4.8, Baden-Württemberg, Germany) and CELENA X (Logos Biosystems, Annandale, VA).

For monitoring the localization of SOD1 recombinant proteins, SOD1-Halo proteins were added in neuronal cultures for 2 h and then the cells were incubated with the cell-permeable HaloTag-fluorscent ligands (diAcFAM) for 30 min. After incubation, cells were washed with serum-free culture medium three times and incubated in serum-containing culture medium in a CO_2_ incubator for 30 min to wash out unbound ligand. Fluorescent images of live cells were obtained using a commercial Tomocube HT-2H holotomography system (Daejeon, Republic of Korea).

Cells with SOD1 aggregation were counted in the fluorescent images. Depending on the intensity of SOD1, cells with SOD1 aggregates were counted in randomly selected fields and quantified as a percentage to the total cells counted. In addition, to calculate the colocalization of the three colors of SOD1, we counted the cells where the three colored images overlapped at a point of high intensity. Cell counting was performed by three independent observers, who were blinded to the transfection or chemical treatment group.

### Transfection of vectors

GFP-SOD1 (WT #26402, G85R #26405, G93A #26406), non-tagged SOD1 (WT #26397, A4V #26398, G37R #26399, G85R #26400, G93A #26401), tdTomato-TDP43 (#28205), mAzurite-C1 (#54583) and mCherry2 (#54563) expression vectors were purchased from Addgene (Cambridge, MA). To generate mAzurite-SOD1-WT and mCherry2-SOD1-WT, the SOD1-WT-expressing cassettes were subcloned into the mAzurite and mCherry2 vectors. SOD1 N53I, D101I and G147P mutations were generated in the pcDNA3.1( +) plasmid by site-directed mutagenesis according to the standard single primer method. Transfection was performed using Jet-PEI reagent or jetOPTIMUS (Polyplus Transfection, New York NY, USA) according to the manufacturer’s protocol. Briefly, the vector was mixed with JetPEI or jetOPTIMUS reagent in 150 mmol/L NaCl buffer or jetOPTIMUS buffer, and incubated for 15 min. The mixture was added to cells in serum-free medium for 4 h. After incubation, the cell medium was replaced with a culture medium supplemented with 10% FBS.

### Microarray analysis

Spinal cord tissue pieces were submerged in the tube containing RNAprotect tissue reagent (Qiagen, Cat. 76104, Hilden, Germany) overnight at lower temperature. Total RNA (500 ng) was extracted from spinal cord tissues of SOD1^G93A−Tg^ mice using an RNAeasy Kit (Qiagen, Cat. 74104). RNA labeling, hybridization on Human Gene 2.0 ST Array (Affymetrix), and data analysis were performed by the DNA Link Company (Gyeonggi-do, Korea). Genes that showed at least a twofold difference were selected for DEG analysis and generating heatmap.

### Measurement of cell viability

Cells were incubated with 0.5 mg/mL of MTT solution (475989; Merck, Darmstadt, Germany) for 4 h at 37 °C. After removing excess solution and washing with PBS, the precipitated materials were dissolved in 200 μL DMSO and quantified by measuring absorbance at 540 nm.

### Chemical synthesis

The compounds (PRG-A-01, 02, 03 and 04) used in this study were synthesized by Dr. Kwon (Seoul National University). The derivatives (PRG-A-02, 03 and 04) were developed based on the core parent structure of PRG-A-01. The brief scheme of synthesis is shown in Figure S1e. More detailed information is available from the corresponding author upon request.

### Statistical analysis

The student’s *t*-test or one-way ANOVA was used for comparisons of two groups. *P* < 0.05 was considered significant. Error bars indicate standard deviation (SD). Data for all figures are expressed as mean ± SD from at least three independent experiments.

## Results

### Optimization of the SOD1 aggregation inhibitor PRG-A-04

**Fig. 1:**
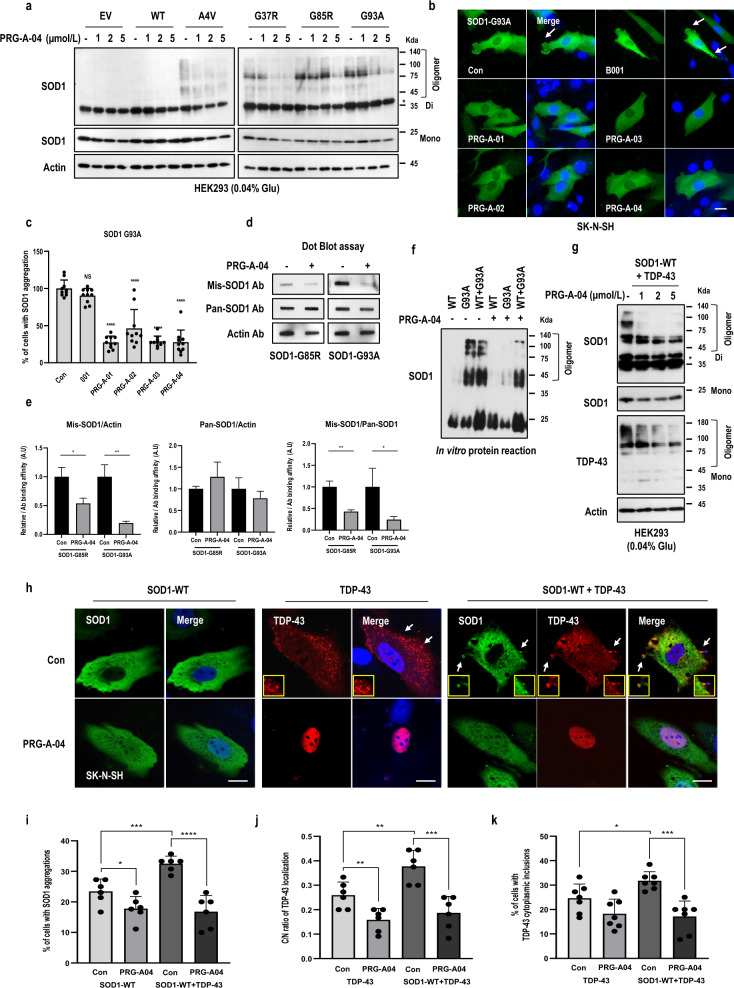
Optimized inhibitor PRG-A-04 reduces SOD1 aggregation. **a** PRG-A-04 suppressed SOD1 oligomerization by MT-SOD1 in a dose-dependent manner, but did not affect dimer formation. **b, c** PRG-A-04 suppressed SOD1 inclusions in SK-N-SH cells. Cells with SOD1 inclusions (white arrows; strong intensity of SOD1) were counted and the percentages are shown with standard deviation. *n* = 3 independent experiments; two-tailed Student’s* t*-test. Scale bar, 10 μm. **d, e** PRG-A-04 reduced misfolding of MT-SOD1. **d** The affinity of misfolding-specific antibody to MT-SOD1 was inhibited by PRG-A-04. **e** Quantification of band intensity with Image J software. Mean ± SD. A.U., arbitrary units. *P < 0.05, **P < 0.01. **f** PRG-A-04 reduced the MT-SOD1 recombinant protein-induced increase of self-oligomerization in vitro. **g** WT-SOD1 and TDP-43 oligomerization induced by ectopic TDP-43 expression was abolished by PRG-A-04 treatment. **h–k** WT-SOD1 aggregation induced by ectopic TDP-43 expression was reduced by PRG-A-04 treatment. PRG-A-04 reduced SOD1 as well as TDP-43 aggregation. **h** SK-N-SH cells were transfected with tdtomato-tagged TDP-43 and/or WT-SOD1 for 24 h. Scale bars, 10 μm. **i** PRG-A-04 reduced WT-SOD1 inclusions triggered by TDP-43 overexpression. Inclusion-positive cells (white arrows in **h**, strong intensity of SOD1) were counted and the percentages are shown as mean ± SD. **j** PRG-A-04 reduced the ratio of cells with cytoplasmic TDP-43 inclusions to tdtomato-positive ones. Cells with cytoplasmic TDP-43 (white arrows) among tdtomato positive ones were counted in randomly selected fields by photomicrographs and the cytosol/nucleus ratio was shown with ± SD. **k** PRG-A-04 reduced cytoplasmic TDP-43 inclusion. Mean ± SD. *P < 0.05, **P < 0.01, ***P < 0.005

We previously found that PRG-A-01 works as an inhibitor of SOD1 aggregation [32], but with poor pharmacokinetics, which requires optimization. In this study, we generated about 20 types of derivatives of PRG-A-01 and tested their inhibitory effects on SOD1 aggregation as well as in vivo therapeutic effects in the ALS mouse model. Among the tested chemicals, 3 chemicals (PRG-A-02, -03 and -04) suppressed the MT-SOD1 aggregation (Figs. 1a–c, S1a–c). Pharmacokinetic analysis showed that the bioavailability of PRG-A-04 (63.78%) was significantly higher than the parent compound or other derivatives (Fig. S1d), so PRG-A-04 was selected for further experiments (Fig. S1e). The PRG-A-04 blocked aggregation of various SOD1 mutants (Figs. 1a, b; S1b) without severe cytotoxicity in normal fobroblast and neuron cell lines (Fig. S1f, g). Since PRG-A-01 could suppress the mis-folding of SOD1 [32], we checked the effect of PRG-A-04 on SOD1 protein folding. Using dot blot analysis with misfolding SOD1-specific antibody, we observed a reduction in misfolding SOD1 with PRG-A-04 treatment (Fig. 1d, e). However, pan-SOD1 antibody binding did not change by PRG-A-04 treatment. These results indicate that PRG-A-04 could resolve the misfolded SOD1 structure to native structure [32, 44, 45]. To validate the ability of PRG-A-04 to inhibit aggregation, we reacted recombinant WT-/MT-SOD1 protein with PRG-A-04 in a non-denaturing condition in vitro. PRG-A-04 inhibited the oligomerizing or aggregation property of MT-SOD1 and the WT/MT-SOD1 co-incubation proteins (Fig. 1f).

We have previously reported TDP-43-related sALS phenotype such as TDP-43 and WT-SOD1 aggregation induced by TDP-43 overexpression [28–31, 33]. Consistent with the PRG-A-01 results, PRG-A-04 indeed exerted an inhibitory effect on TDP-43 and SOD1 aggregation (Fig. 1g–i). Moreover, cytosolic mislocalization of TDP-43, a characteristic feature of pathological TDP-43-associated sALS, was significantly reduced by treatment with PRG-A-04 (Fig. 1j, k). These results suggest that PRG-A-04 would be applicable to both sporadic and familial types of ALS.

### PRG-A-04 targets trimeric SOD1 to inhibit SOD1 aggregation

**Fig. 2:**
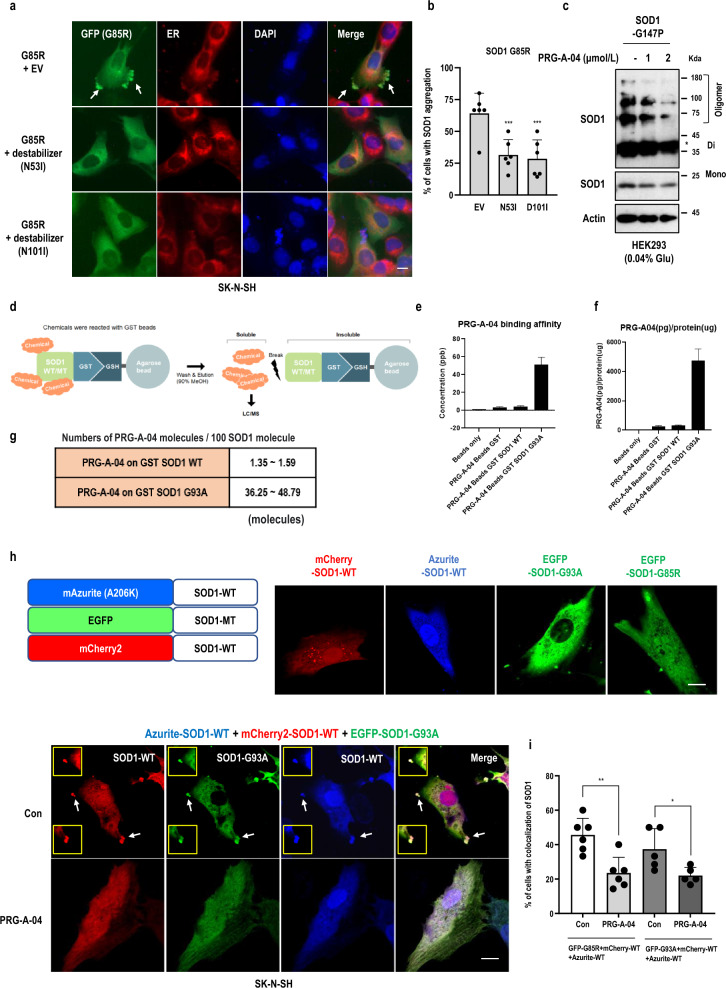
PRG-A-04 suppresses SOD1 aggregation through targeting trimeric SOD1. **a**-**b** SOD1 trimer destabilizer inhibited MT-SOD1 aggregation. (**a**) SOD1 (GFP) expression was observed under fluorescence microscope (white arrows; strong intensity of SOD1). Scale bar, 10 μm. EV, empty vector. (**b**) SOD1 inclusion-positive cells (white arrows; strong intensity of SOD1) were counted and the percentages are shown as mean ± SD. **c** PRG-A-04 reduced SOD1 oligomerization induced by SOD1 trimer stabilizer (G147P). **d-g** PRG-A-04 directly bound to the mutant form of SOD1. (**d**) Scheme of GST-Pull down assay to confirm binding affinity between PRG-A-04 and SOD1 proteins. (**e**) PRG-A-04 showed a stronger binding affinity to MT-SOD1 than to WT-SOD1 protein. (**f**) The PRG-A-04/protein ratio was high in the sample that reacted with the GST-MT-SOD1 recombinant protein. (**g**) For quantitative analysis, the number of PRG-A-04 molecules per 100 SOD1 molecules was counted. PRG-A-04 could directly bind to mutant SOD1 in a ratio of approximately 1:3. **h, i** PRG-A-04 abolished co-inclusions of three different color variants of SOD1. (**h**) Cells were transfected with WT-SOD1 (Azurite and mCherry2) and MT (G93A)-SOD1 (GFP) vectors and treated with PRG-A-04 (5 μmol/L) for 24 h. SOD1 expression was observed under Apotome microscope (white arrows; strong intensity and co-localization of SOD1). Scale bar, 10 μm. (**i**) Cells with WT and MT-SOD1 co-inclusions were counted and the percentages are shown as mean ± SD. For cell counting, five fluorescence images were randomly selected. Cell counting was performed by three independent observers, who were blinded to transfection or chemical treatment group. *P < 0.05, **P < 0.01, ***P < 0.005

Next, we wondered how PRG-A-04 reduces the SOD1 aggregation complex. Due to the difficulty in interpreting the SOD1 aggregation/misfolding process, the formation of the cytotoxic SOD1 protein structure remains unknown. Recently, it has been reported that the SOD1 trimer formation is important for SOD1 aggregation [39–43]. To verify this, we constructed SOD1 trimer-destabilizing mutant expression vectors (SOD1-N53I, D101I). We found that the SOD1 aggregation/oligomerization caused by the pathological MT-SOD1 (G85R, G93A) overexpression was diminished by the trimer-destabilizing mutants (Figs. 2a, b; S2a). Conversely, the SOD1 aggregation/oligomerization was induced by the SOD1 trimer-stabilizing mutant SOD1-G147P (Fig. 2c). These results indicate that SOD1 aggregates are stably formed based on the trimeric SOD1 structure. Furthermore, PRG-A-04 reduced the SOD1 oligomerization caused by the trimer-stabilizing mutant overexpression (Fig. 2c). Therefore, we next sought to determine whether the aggregation-inhibitory effect of PRG-A-04 is related to the trimeric structure of SOD1.

In our previous study, we isolated PRG-A-01 (Hit compound) through an enzyme-linked immunosorbent assay-based drug screening system for inhibition of WT-MT SOD1 binding. PRG-A-01 did not show inhibitory effects on WT-WT SOD1 interaction or bind to the surface of the dimeric structure of native SOD1 protein [32]. We assumed that PRG-A-04 inhibits the aggregation by directly binding to the unknown specific structure that is exposed during SOD1 aggregation. To test our hypothesis, GST-conjugated SOD1 recombinant protein and PRG-A-04 were incubated at room temperature. The mixtures were separated into soluble and insoluble fractions by centrifugation and eluted with Me-OH. LC–MS/MS was performed on the soluble fraction for confirming interaction between PRG-A-04 and SOD1 protein (Fig. 2d). Interestingly, PRG-A-04 showed a stronger binding affinity to MT-SOD1 than to WT-SOD1 protein (Fig. 2e, f). We calculated the number of PRG-A-04 molecules per 100 SOD1 molecules and revealed that PRG-A-04 could directly bind to MT-SOD1 in a ratio of approximately 1:3 (Figs. 2g and S2b). Based on these results, we inferred that PRG-A-04 directly reacts with the trimeric form of SOD1 at a relatively high rate. To check the possibility of the formation of trimeric SOD1 by MT-SOD1 overexpression, we constructed SOD1 vectors expressing three different color variants and co-transfected the encoding vectors into SK-N-SH cells. The diffused WT-SOD1 (Azurite and mCherry2) was aggregated by MT-SOD1 (GFP) and condensed together at one spot (Figs. 2h, i; S2c). We also observed that the co-inclusion of WT and MT-SOD1 disappeared upon treatment with PRG-A-04 (Figs. 2h, i; S2c). These results suggest that MT-SOD1 induces the accumulation of WT-SOD1 and that the trimeric structure is formed during this process. In addition, PRG-A-04 may resolve SOD1 aggregation by targeting the trimeric SOD1 structure. Interestingly, three different-color variants of SOD1-WT (Azurite, mCherry2 and GFP) were co-localized when the vectors were co-transfected into neuronal cells and exposed to cellular stresses such as low pH and presence of Ca^2+^ chelator (Fig. S2d, e). We speculate that the trimeric SOD1 structure might be formed in both mutant (fALS) and physiological (sALS) conditions and drives SOD1 aggregation. PRG-A-04 inhibits SOD1 aggregation possibly by targeting the SOD1 trimeric structure during the nucleation phase of aggregation.

### SOD1 propagation through trimeric SOD1 formation in neurons

**Fig. 3:**
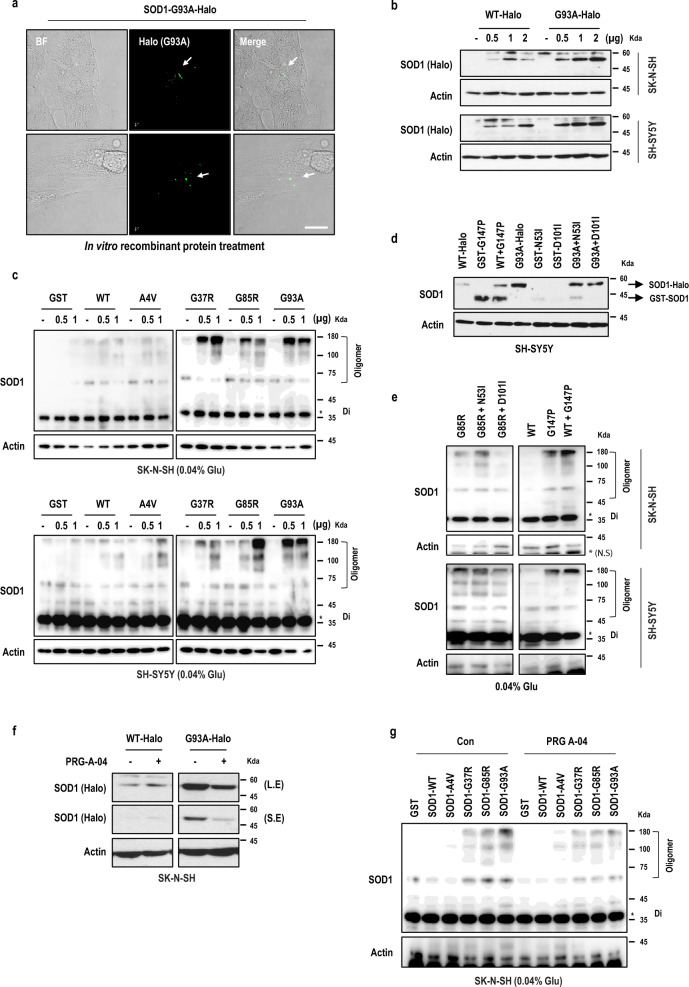
Mutant SOD1 propagation through trimeric SOD1 formation. **a** In vitro SOD1 recombinant proteins were localized intracellularly in neurons. White arrows indicate the intensity of SOD1. BF; Bright field. Scale bar, 400 μm. **b** The uptake of MT-SOD1 was more efficient than that of WT-SOD1 in neurons. **c** The high uptake of MT-SOD1 protein induced SOD1 oligomerization in neuronal cells. **d** The uptake efficiency in the presence of SOD1 trimer stabilizer (G147P) was higher, similar to that in the presence of MT-SOD1 (G93A) protein. The low uptake efficiency of WT-SOD1 was increased by the trimer stabilizer. **e** WT-SOD1 oligomerization was accelerated by the uptake of SOD1 trimer stabilizer. On the other hand, the SOD1 oligomerization induced by MT-SOD1 (G85R) protein was abolished by SOD1 trimer destabilizer (N53I, D101I). N.S., non-specific band. **f** PRG-A-04 selectively inhibited MT-SOD1 uptake in neurons. **g** PRG-A-04 reduced SOD1 oligomerization induced by SOD1 protein uptake

Although the pathological hallmark of ALS disease is SOD1 spreading across the synapses to surrounding neurons, the exact mechanism of SOD1 spreading is unknown [24, 35–38]. We hypothesized that pathological SOD1 might propagate more than WT-SOD1, leading to progressive neurodegenerative disease. To test the ability of SOD1 to be taken up by neuronal cell lines, we prepared SOD1-Halo and SOD1-GST recombinant proteins and added them in neuronal cultures (Figs. 3a, S3a, b). The SOD1 protein uptake was inhibited by an endocytosis inhibitor Dynasore [46], suggesting that SOD1 propagation is achieved by endocytosis through intracellular connections (Fig. S3a). Next, to check the localization of SOD1 proteins, we monitored them using the HaloTag Fluorescent Ligands (cell permeable ligands, diAcFAM) in live neuronal cells. The MT-SOD1 proteins were present intracellularly under non-permeable conditions (Figs. 3a and S3b). These results indicate that the uptake of MT-SOD1 proteins is achieved by endocytosis. Indeed, the uptake of MT-SOD1 proteins was also more efficient than WT-SOD1 in neurons (Fig. 3b). We speculated that SOD1 aggregates would be formed by the uptake of SOD1 protein. Actually, the degree of SOD1 oligomerization depends on the efficiency of SOD1 uptake and consistently, our results showed that SOD1 oligomerization was accelerated upon exposure to MT-SOD1 proteins (Fig. 3c).

Next, to determine the effect of the trimeric structure of SOD1 on protein uptake efficiency, we constructed the SOD1 trimer-stabilizing and destabilizing mutant proteins. Interestingly, the uptake of the SOD1 trimer-stabilizing mutant G147P was more efficient than that of the trimer-destabilizer mutants N53I and D101I (Fig. 3d). Additionally, co-treatment with the trimer-stabilizing mutant and WT-SOD1 proteins increased the level of WT-SOD1 protein uptake. On the other hand, the high uptake efficiency of MT-SOD1 (G93A) was decreased in the presence of SOD1 trimer-destabilizer mutant proteins (Fig. 3d). The MT-SOD1 (G85R) oligomerization was abolished by co-treatment with trimer-destabilizer mutant proteins (Fig. 3e). These results indicated that the SOD1 trimer formation is important for SOD1 protein uptake.

We hypothesized that PRG-A-04, which targets trimeric SOD1, would inhibit SOD1 protein uptake. MT-SOD1 proteins were incubated with PRG-A-04 in vitro, and then cells were treated with the mixture. The MT-SOD1 protein uptake was reduced by incubation with PRG-A-04, as was SOD1 oligomerization (Fig. 3f, g). Thus, PRG-A-04 not only reduces SOD1 aggregation in the intracellular compartment, but also inhibits SOD1 propagation in the extracellular environment by targeting trimeric SOD1. However, whether neuron-specific receptors on synapses, physiological conditions (neuronal cellular stress such as ion deficiency, environmental conditions such as hypoxia) and other ALS-related genetic causes (TDP-43, FUS, etc.) are essential for SOD1 uptake requires further study.

### In vivo effect of PRG-A-04 on *SOD1* G93A transgenic mice

**Fig. 4:**
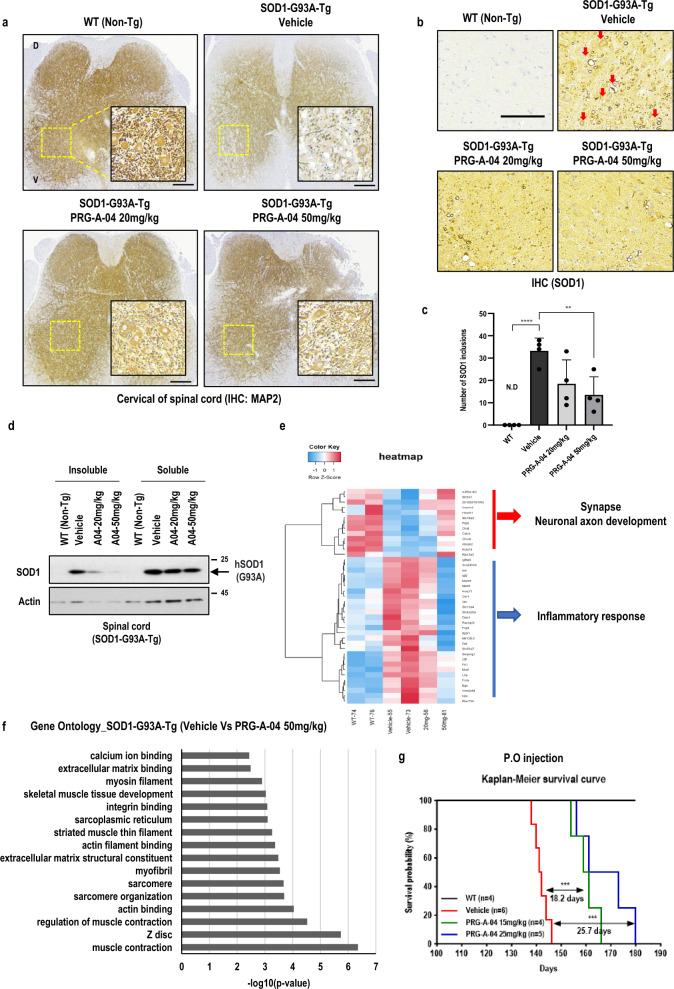
PRG-A-04 ameliorates ALS progression in the SOD1^G93A−Tg^ mouse model. **a** The neuronal markers were maintained in mice receiving i.p. injection of PRG-A-04. Low expression of MAP2 was detected in vehicle (DMSO)-treated control mice. Representative images are shown at a magnification of 10 × , with inserts of 20 × (yellow box). Enlarged pictures of cervical and lumbar spinal cords are shown in Fig. S4g and S4i. The intensity of neuronal markers was counted and plotted in Fig. S4h and S4j. Scale bar, 20 μm. **b** Compared to DMSO-treated 17-week-old mice (Con, *n* = 4), PRG-A-04 reduced SOD1 aggregation with SOD1-positive vacuoles (red arrowheads) (20 mg/kg, *n* = 3, 50 mg/kg, *n* = 3). Scale bar, 20 μm. **c** Number of SOD1 inclusions was counted with SOD1-positive vacuoles. N.D., not detectable. **P < 0.01, ****P < 0.001. **d** The insoluble SOD1 was reduced by PRG-A-04 in the cervical spinal cord tissue lysates of mice carrying SOD1-G93A mutation. **e** The neuronal protective effect of PRG-A-04 was confirmed by microarray in spinal cord tissue. **f** Gene ontology analysis of spinal cord tissue between the vehicle- and PRG-A-04-treated SOD1^G93A−Tg^ mice. **g** Kaplan Meier survival curve of SOD1^G93A−Tg^ ALS mice. Compared to vehicle-treated mice (Con, *n* = 6), PRG-A-04 treatment by p.o. injection at 15 mg/kg (*n* = 4) and 25 mg/kg (*n* = 5) extended the survival by about 18 and 25 days, respectively. ***P < 0.005

PRG-A-04 (20 mg/kg or 50 mg/kg) was injected intraperitoneally (i.p., twice a week) in 11-week-old SOD1^G93A−Tg^ mice to verify its efficacy in vivo (Fig. S4a). PRG-A-04 showed no cytotoxicity regarding body weight loss (Fig. S4b). We performed the rotarod test to monitor muscle strength. After training for 2 weeks from the age of 9 weeks, the latency to fall from the rotarod at 11 weeks (initiation of PRG-A-04 injection), 14 weeks (injection for 4 weeks) and 16 weeks (injection for 6 weeks) was recorded. The latency to fall at 16 weeks was decreased by ~ 43% compared to that at 11 weeks in the vehicle-treated group, while in the PRG-A-04 groups, the latency was reduced by 14.4% and 20.1% at 16 weeks, respectively, similar as the wild-type animals (Fig. S4c). Histological analysis at 16 weeks revealed that PRG-A-04 increased the number of neurons in cervical region of the spinal cord compared to the vehicle-treated mice (Fig. S4d, e). This neuron-protective effect of PRG-A-04 was confirmed using neuron-specific markers MAP2 (Fig. 4a, Fig. S4g, h) and NeuN (Fig. S4i, j).

Our previous results confirmed the therapeutic efficacy of the drug by reducing SOD1 inclusion bodies with vacuolar structures in an ALS mouse model [32]. Here, immunohistochemistry showed that PRG-A-04 reduced SOD1 inclusion bodies (Fig. 4b, c), and western blotting confirmed the decrease of insolubility and oligomerization of SOD1 (Figs. 4d and S4f). Interestingly, even at age of 18 weeks when accelerated motor dysfunction phenomenon was observed, PRG-A-04 significantly improved motor function (Fig. S5a–c). The total distance travelled and the speed of walking were significantly improved by PRG-A-04. Consistently, the neurodegenerative features were alleviated by PRG-A-04 as revealed by staining for neuronal markers (Fig. S5d–f). To further analyze the efficacy of PRG-A-04 on neuronal protection, we performed microarray using spinal cord tissue and obtained two important findings (Fig. 4e). First, genes related to neuronal axon development and synapse stability were downregulated in the vehicle-treated mice, and the down-regulation was rescued by PRG-A-04 in a dose-dependent manner. In neurodegenerative diseases, neuronal networks are disrupted by synaptic failure [47–49]. Synaptic stability is primarily regulated by neurotransmitters, Ca^2+^ ion balance, and cytoskeletal stability [47–50]. Therefore, stable synaptic formation is important for maintaining neuronal networks and is an important measure in the treatment of neurodegenerative diseases. Second, genes associated with neuro-inflammatory response were upregulated in the vehicle-treated mice, and the up-regulation was rescued by PRG-A-04. Next, ontology analysis identified gene sets related to motility, including muscle contraction, actin binding, sarcomere, Ca^2+^ ion binding and inflammatory response, that were regulated by PRG-A-04 (Figs. 4f and S6a). KEGG pathway analysis also indicated that pathways related to motility, such as calcium signaling, protein digestion, and regulation of the actin cytoskeleton, were regulated by PRG-A-04 (Fig. S6b).

In order to reduce the stress of injection and increase the efficacy of the drug through continuous drug injection into the body, mice were fed daily from an early stage (7 weeks), accompanied by i.p. injection starting at 11 weeks (Fig. S7a, b). Similar to previous results (Fig. S4), PRG-A-04 maintained the motility (Vehicle group, about 80% reduction; PRG-A-04 group, 50% reduction at 18 weeks) and the body weights of ALS mice (Fig. S7c, d). Recorded behaviors in the open-field test are shown in Supplementary videos 1–3. In addition, the combined PRG-A-04 treatment extended survival by about 25–30 days compared to other PRG-A-series of derivatives (Fig. S7e). Based on these results, we assumed that prolonged drug retention in the body could improve drug efficacy.

**Fig. 5:**
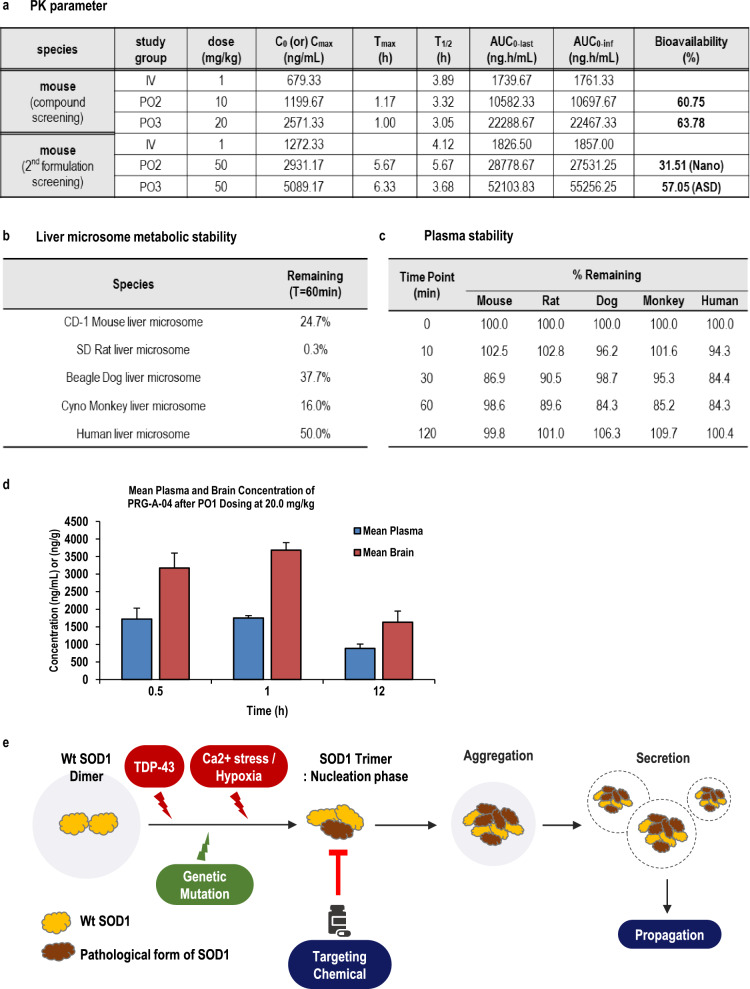
Preclinical data of PRG-A-04. **a** The pharmacokinetics (PK) profiling indicated that the PRG-A-04 compound has a bioavailability of about 60% in mice. After formulation development, PRG-A-04 administered orally (ASD, Amorphous solid dispersions) showed about 57% bioavailability. C_max_ (ng/mL), maximum blood concentration; T_max_ (h), time of peak blood concentration; AUC; area under the curve (*t* = 0 to infinite). **b** PRG-A-04 was moderately metabolized in CD-1 mouse, beagle dog, cynomolgus monkey, and human liver microsomes, and highly metabolized in SD rat liver microsomes. **c** PRG-A-04 was stable in CD-1 mouse, SD rat, beagle dog, cynomolgus monkey and human plasma after 120 min of incubation. **d** Plasma and brain concentrations of PRG-A-04 after p.o. dosing at 20 mg/kg. PRG-A-04 showed the brain/plasma ratio about 2.11. **e** Illustration of SOD1 aggregation through SOD1 trimer formation in the presence of genetic mutation (fALS) and cellular stresses (sALS), and the working mode of PRG-A-04 to inhibit SOD1 trimerization

Next, through formulation development, we developed a drug to be administered orally to improve convenience and compliance for ALS patients who have limited mobility and are physically burdened by medication. We developed an ASD formulation with an approximately bioavailability of ~ 57%. Efficacy testing was conducted through oral administration (p.o. injection) twice a week from 9 weeks (Figs. 5a, S7f). We demonstrated that oral administration of PRG-A-04 exhibited drug efficacy, including movement preservation (vehicle group, about 73% reduction; PRG-A-04 group, 45% reduction at 17 weeks) and lifespan extension for about 25.7 days, without toxicity, thereby confirming the possibility of developing an oral agent for the treatment of ALS (Fig. S7g, h; Fig. 4g). Recorded behaviors in the open-field test are shown in Supplementary videos 4 and 5.

### In vitro ADME (absorption, distribution, metabolism, excretion) analysis shows druggable property of PRG-A-04

Next, we checked the basic in vitro ADME test including pharmacokinetics, plasma protein binding, CYP (Cytochrome P450) inhibition, metabolic stability and blood–brain barrier (BBB) permeability. The oral administration formulation ASD showed favorable bioavailability (~ 57%) in mice (Fig. 5a). PRG-A-04 was metabolized significantly in liver microsomes of CD-1 mice, beagle dogs, and cynomolgus monkeys, but moderately in human liver microsomes (Fig. 5b). Also, PRG-A-04 was stable in the plasma of CD-1 mice, SD rats, beagle dogs, cynomolgus monkeys and humans after 120 min of incubation (Fig. 5c). Furthermore, PRG-A-04 exhibited a high binding affinity to plasma protein in the five species with no significant difference between species (Fig. S8a). From these analysis, we did not find severe cytotoxic effect of PRG-A-04 (Fig. S8a, b). We also plan to test safety panel assay in the future to obtain safety data for the drug. To confirm the BBB permeability of PRG-A-04 for the development of ALS treatment, parallel artificial membrane permeability assay (PAMPA) and mouse brain tissue penetration assay were performed. The BBB-PAMPA test revealed that PRG-A-04 had moderate permeability. The mouse brain tissue penetration experiment with p.o. injection (20 mg/kg) showed that PRG-A-04 passed the BBB with a brain/plasma ratio of 2.11 (Figs. 5d; S8c, d). These results indicate that PRG-A-04 has sufficient drug properties for future clinical development, suggesting the potential of successful therapeutic development.

## Discussion

In our previous studies, we have shown the therapeutic effect of PRG-A-01 through inhibiting SOD1 aggregation induced by *SOD1* mutation, TDP-43 overexpression and cellular stresses [32]. Despite its neuronal protective and life-extending effects in SOD1^G93A−Tg^ ALS mice, PRG-A-01 is poorly bioavailable and rapidly degraded in vivo. In this study, to overcome these limitations, we generated the derivatives by changing the linker domain or the side chain of PRG-A-01 and finally obtained the most plausible drug candidate, PRG-A-04. PRG-A-04 exhibited favorable pharmacological properties as a therapeutic agent, including high bioavailability and BBB penetration (Figs. 5 and S8). We confirmed the therapeutic effect of PRG-A-04, which inhibited SOD1 aggregation by binding to trimeric SOD1, disrupting its large aggregated complex (Fig. 2). In addition, PRG-A-04 decreased insoluble form of SOD1, protected against neuron loss in the spinal cord, maintained movement, and extended life span of ALS model mice (Figs. 4 and S7). To pave the way for clinical studies in ALS patients, PRG-A-04 was further developed as an oral administration drug to improve the convenience and compliance of ALS patients. The PK profile suggests that delivery through endotracheal intubation or intravenous injection may also be useful for patients with advanced disease who have difficulty swallowing. Oral administration of PRG-A-04 demonstrated life-extending efficacy. Surprisingly, when administered through feeding in combination with i.p injection, PRG-A-04 maintained the body weight of ALS mice, in contrast to the vehicle group which showed a loss of body weight (Figs. 5 and S7). Therefore, we assume that the efficacy may be improved if the drug stays in the body for a longer period, and we are conducting further studies to improve the efficiency of drug delivery in the body. In addition, compared to Tofersen, which requires intrathecal injection, PRG can be developed as an orally administered drug and is expected to be valuable as a future ALS treatment.

Currently, there are four FDA-approved drugs for the treatment of ALS, including riluzole (GABA receptor inhibitor), radicava (antioxidant), tofersen (SOD1-ASO), and relyvrio (coformulation of sodium phenylbutyrate and taurursodiol) [9–17]. However, riluzole and radicava have shown efficacy in delaying ALS progression, but no efficacy on survival rate [9–12]. Relyvrio (chaperone and inhibitor of mitochondrial-associated apoptosis) received conditional FDA approval, but was withdrawn in April 2024 due to the lack of efficacy in phase 3 clinical trials [15, 16, 18]. Tofersen, the first treatment targeting the mutant *SOD1* gene, failed to improve functional decline in clinical trials, but received conditional approval from the FDA based on reductions of surrogate markers and cerebrospinal fluid neurofibrillary tangle (NFL) compared to patients receiving a placebo. This suggests that tofersen may have disease-modifying effects in some SOD1-ALS patients, but definitive therapeutic efficacy remains to be validated in further clinical trials [14, 17, 51]. Among the four drugs approved so far, tofersen, which directly targets the *SOD1* gene, has been reported to be most effective. In addition, targeting misfolded or oligomeric SOD1 proteins has been reported to improve neuropathology [45, 52, 53]. These reports suggest that the SOD1 protein is a valid target for ALS treatment.

Abnormal protein aggregates such as SOD1, TDP-43, and C9orf72 have long been studied [5–7]. However, it is still unknown how abnormal protein aggregation is formed, which makes it difficult to interpret the pathogenesis of ALS and develop treatments. Recent reports have shown that the trimer structure formation is important for aggregation [39–43]. Here, we confirmed that aggregation of SOD1, which can be caused by mutant SOD1, was inhibited with a trimer destabilizer SOD1 (Fig. S2). In addition, three-color WT-SOD1 formed co-inclusions under cellular stresses such as low pH and Ca^2+^ chelator (Fig. S2d, e). In addition, we found that the formation of the trimer structure is important for the propagation of the SOD1 protein (Fig. 3). These results suggest that the trimeric SOD1 structures are formed during aggregation and propagation of SOD1 proteins, both in mutation conditions (fALS) and under physiological conditions (sALS).

In this study, PRG-A-04 exhibited an inhibitory effect on MT-SOD1 aggregation (Fig. 1), particularly on aggregation by SOD1 stabilizer SOD1-G147P (Fig. 2). Furthermore, PRG-A-04 inhibited the uptake of MT-SOD1, thereby inhibiting SOD1 oligomerization. Also, we speculate that PRG-A-04 may bind to the trimeric structure of SOD1, inhibit the binding of TDP-43 to SOD1, thereby inhibiting TDP-43-induced SOD1 aggregation. We have previously confirmed that PRG-A-01 does not react with the surface of the dimer structure or interfere with SOD1 dimer formation [32]. Here PRG-A-04 produced the same results. In this study, we confirmed that PRG-A-04 binds specifically to the MT-SOD1 protein at a ratio of approximately 1:3 using a GST-pull down assay and LC–MS/MS (Fig. 2). This implies that PRG-A-04 may directly interact with the trimeric structure exposed upon SOD1 aggregation, although more detailed studies are needed. Based on these results, we speculate that PRG-A-04 inhibits SOD1 protein aggregation by binding to the early aggregates, thereby preventing the formation of large complexes, rather than inhibiting large complexes that have already formed (Fig. 5e). Currently, we are conducting modeling studies on the SOD1 trimer structure to determine how SOD1 aggregates are formed from SOD1 trimer, how the aggregates bind to the TDP-43 protein, and to which domain of the SOD1 trimer the PRG-A-04 binds.

It has been reported that 97% of ALS patients exhibit TDP-43 alterations [31]. However, it is unclear whether the pathology associated with TDP-43 is a gain of toxicity (gain of function) due to TDP-43 aggregation in the cytosol or a loss of function (LOF) due to mislocalization in the cytosol [54–58]. It has been reported that mislocalization of nuclear TDP-43 in the cytosol causes RNA instability. As a result, abnormalities occur in the encoding process of the *STMN2* and *UNC13A* genes, leading to the abnormal inclusion of cryptic exons. As STMN2 and UNC13A play a crucial role in axon stability and regeneration in neurons, dysregulation may contribute to the progression of ALS [59–63]. Many studies have reported that TDP-43 cytoplasmic inclusions in neurons and glial cells are a key pathological feature of ALS and frontotemporal dementia [33, 64]. TDP-43 amplification and mutations have been reported to cause aggregation of TDP-43 in the cytoplasm. This aggregation disrupts the formation of stress granules, which are important for protecting cells under stress. As a result, TDP-43 aggregation causes neuronal cell toxicity as a failure of defense mechanisms [54–56, 58]. In addition, SOD1 proteins are colocalized with TDP-43 aggregates in many sALS patients, suggesting that SOD1 pathology is caused by TDP-43 protein aggregation [28–31, 33]. Here we confirmed that TDP-43 overexpression leads to cytoplasmic mislocalization and co-aggregation of SOD1 with TDP-43 protein in the cytoplasm (Fig. 1e-i). We are investigating whether cytoplasmic TDP-43 is associated with the trimeric SOD1 structure. In addition, we found that PRG-A-04 significantly reduced TDP-43 self-oligomerization (Fig. 1e). PRG-A-04 also decreased cytoplasmic TDP-43 (Fig. 1f, h, i). Based on these results, we hypothesize that resolving the TDP-43 aggregation would allow TDP-43 to re-enter the nucleus and exert its function. Therefore, we speculate that PRG-A-04 may also have therapeutic efficacy for LOF through repositioning TDP-43 to the nucleus.

## Conclusions

In summary, we demonstrate that targeting trimeric SOD1 with PRG-A-04 can effectively inhibit the uptake of MT-SOD1 and ameliorate abnormal SOD1 aggregation induced by expression of MT-SOD1, TDP-43 and cellular stress. PRG-A-04 protects against motor neuron loss, preserves muscle strength and extends the lifespan of ALS mouse model. Our results suggest that PRG-A-04 may be a promising candidate for both sporadic and familial ALS patients.

## Supplementary Information

**Additional file 1. Supplementary Video #1**: Video of 19-week-old SOD1^G93A−Tg^ female mice treated with PRG-A-04 through feeding combined with i.p. injection. Compared to the vehicle-treated mice (Supplementary video #3), PRG-A-04 maintained the movement ability

**Additional file 2. Supplementary Video #2**: Video of 19-week-old SOD1^G93A−Tg^ female mice treated with PRG-A-04 through feeding combined with i.p. injection. Compared to the vehicle-treated mice (Supplementary video #3), the PRG-A-04-treated mouse still showed movement ability, despite some problem in hind leg movement.

**Additional file 3. Supplementary Video #3**: Video of 19-week-old SOD1^G93A−Tg^ female mice treated with vehicle, which showed paralysis of hind legs and impaired movement.

**Additional file 4. Supplementary Video #4**: Movie of 19-week-old SOD1^G93A−Tg^ female mice. The vehicle-treated mouse laid down and could not move. In contrast, the other two mice (PRG-A-04-treated) were moving continuously.

**Additional file 5: Fig. S1**. Optimized inhibitor reduces SOD1 aggregation. **Fig. S2**. Mutant SOD1 induces aggregation through trimeric SOD1 formation. **Fig. S3**. SOD1 protein uptake into the cells through endocytosis. **Fig. S4**. Therapeutic effect of PRG-A-04 through i.p. injection on the SOD1^G93A−Tg^ mouse. **Fig. S5**. PRG-A-04 maintains the movement disorder on the SOD1G^G93A−Tg^ mouse model. **Fig. S6**. PRG-A-04 improves muscle contraction related gene expression. **Fig. S7**. PRG-A-04 extended the lifespan of SOD1^G93A−Tg^ mice through oral administration. **Fig. S8**. Preclinical data of PRG-A-04.

**Additional file 6**. Raw data.

**Additional file 7. Supplementary Video # 5**: Movie of a 19-week-old WT female mouse, which showed normal movement.

## Data Availability

All data generated in this study are included in this published article. Mouse behavior datasets are shown in Supplementary video files. Raw datasets during the current study are available from the corresponding author upon request.

## Abbreviations

ALS: Amyotrophic lateral sclerosis
NDD: Neurodegenerative disease
AD: Alzheimer’s disease
MT: Mutant-type
sALS: Sporadic ALS
ASD: Amorphous solid dispersions
BBB: Blood–brain barrier
PAMPA: Parallel artificial membrane permeability assay
NFL: Neurofibrillary tangle
LOF: Loss of function
