# Supplementary material for: Inhibition of SOD1 trimerization is a novel drug target for ALS disease
Source: Transl Neurodegener. 2025 May 12;14:21. doi: 10.1186/s40035-025-00483-8 (PMC12067741; doi:10.1186/s40035-025-00483-8)
Supplement: Supplementary file 5 — Additional file 5: Fig. S1. Optimized inhibitor reduces SOD1 aggregation. Fig. S2. Mutant SOD1 induces aggregation through trimeric SOD1 formation. Fig. S3. SOD1 protein uptake into the cells through endocytosis. Fig. S4. Therapeutic effect of PRG-A-04 through i.p. injection on the SOD1G93A−Tg mouse. Fig. S5. PRG-A-04 maintains the movement disorder on the SOD1GG93A−Tg mouse model. Fig. S6. PRG-A-04 improves muscle contraction related gene expression. Fig. S7. PRG-A-04 extended the lifespan of SOD1G93A−Tg mice through oral administration. Fig. S8. Preclinical data of PRG-A-04. [file 40035_2025_483_MOESM5_ESM.pdf]

Fig. S1. Optimized inhibitor reduces SOD1 aggregation

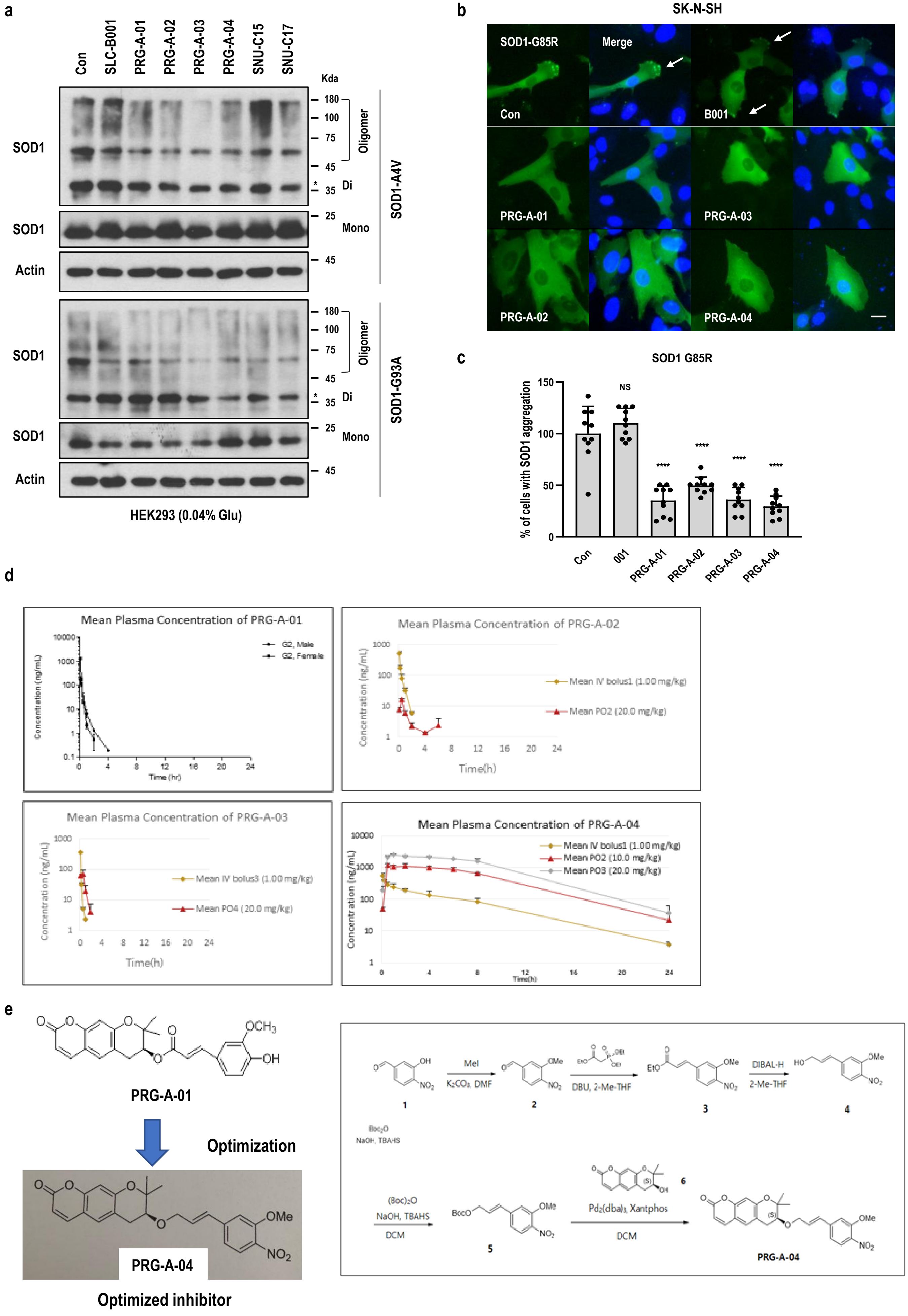

Fig. S1. Optimized inhibitor reduces SOD1 aggregation

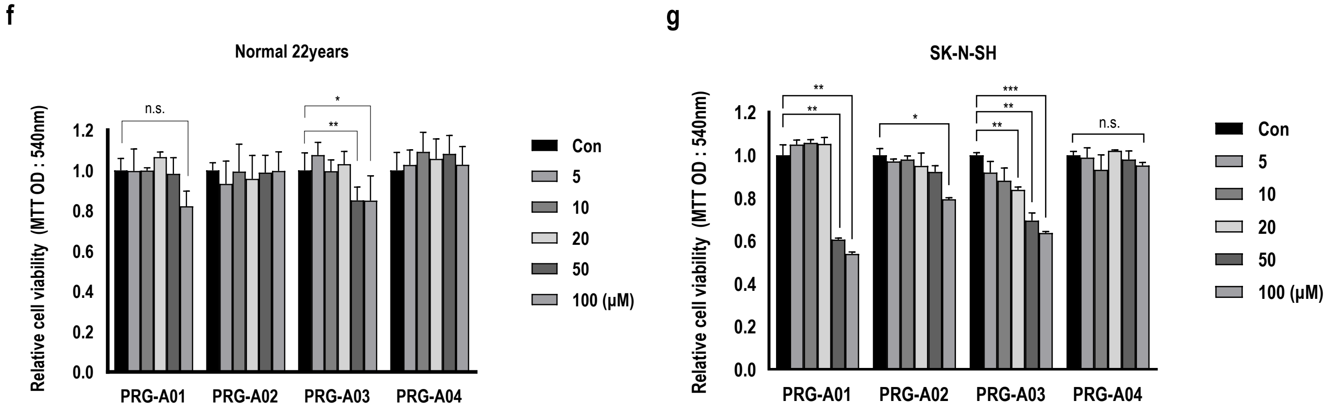

Fig. S1. Optimized inhibitor reduces SOD1 aggregation. a, Mutant SOD1s induced WT-SOD1 aggregation. Testing the selected chemicals to check the inhibitory effect on SOD1 aggregation. HEK293 cells were transfected with MT-SOD1 vectors (A4V, G93A) and treated with selected chemicals (5 μM). After incubation, transfected cells were harvested with TNNI buffer and fixed with 0.04% glutaraldehyde (Glu) for 30min. Samples were separated using SDS-PAGE. **b-c,** PRG-A-04 inhibited SOD1 inclusions in SK-N-SH cells. (b) Cells expressing MT-SOD1 vectors were treated with PRG-A-04 (5 μM) for 24 hr. After incubation, cells were fixed with 4 % PFA and observed under the fluorescence microscope. (c) Cells with SOD1 inclusions (white arrows; strong intensity of SOD1) were counted by photomicrographs and the percentages were shown with standard deviation (SD). For cell counting, ten fluorescence images were randomly selected. Counting of cells were performed by three independent observers, who were blinded to transfection or chemical treatment group; two-tailed Student's t-test. NS; Not significant, ****$P < 0.001$, **d,** PRG-A-04 showed the improved bioavailability. Pharmacokinetics analysis of PRG-A-01,02 and 03 indicated that the rapid degradation in blood as well as gastro-intestinal tract. IV; intravenous injected group, PO; Per Os administrated group **e,** The structure of optimized chemical, PRG-A-04 (left) and brief synthetic scheme of PRG-A-04. **f-g,** PRG-A-04 did not show cytotoxicity. To know the toxic effect of PRG-A-04, human normal fibroblast (f) and neuron cells (g) were incubated with PRG-A-04 for 48 hr dose dependently and measured the viability through MTT assay. "Con" means Dimethyl sulfoxide (DMSO)-treated control. The data are normalized to DMSO-treated cells. n=3 independent experiments; two-tailed Student's t-test. NS; Not significant, *$P < 0.05$, **$P < 0.01$, ***$P < 0.005$.

Fig. S2. Mutant SOD1 induces aggregation through trimeric SOD1 formation

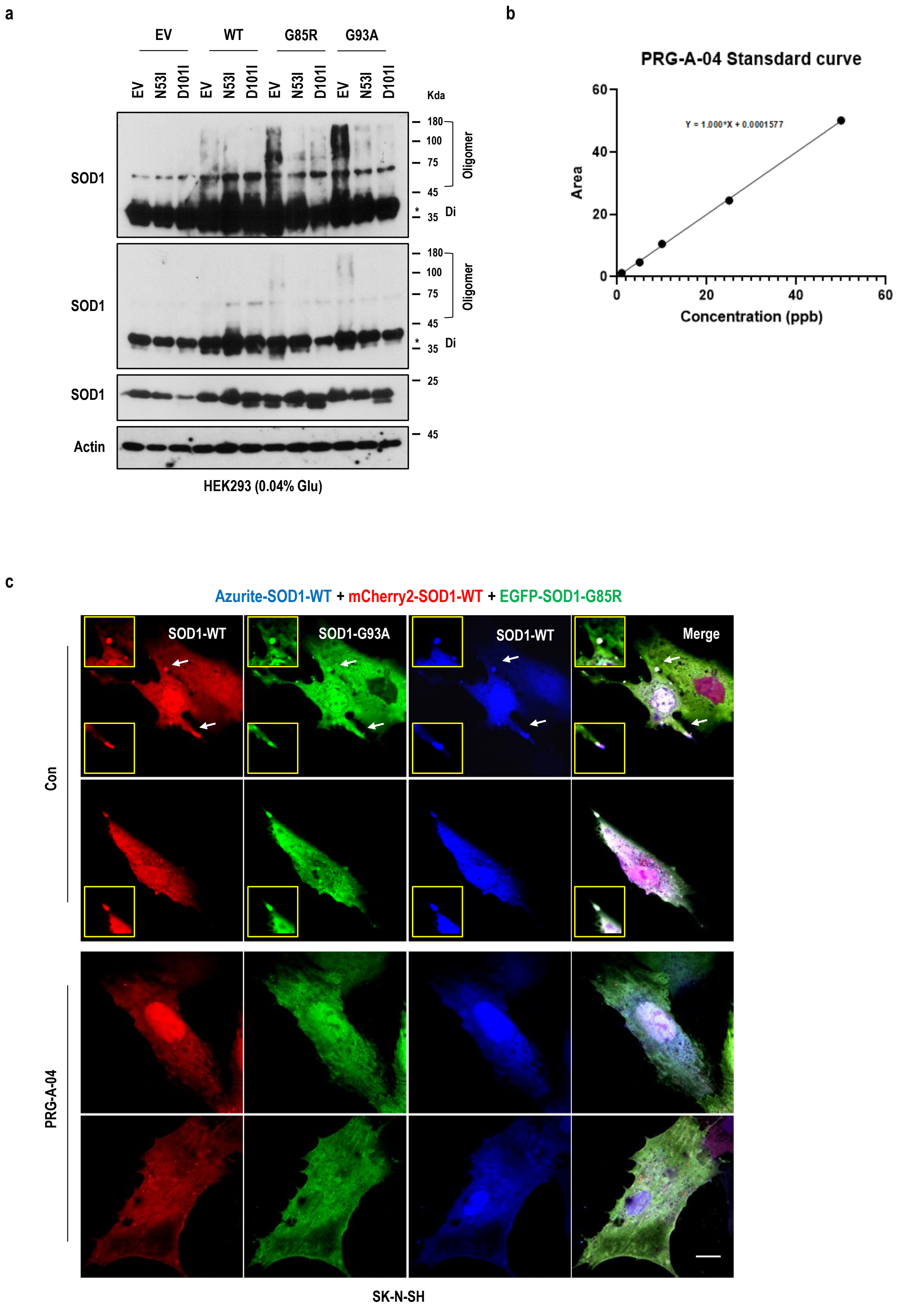

Fig. S2. Mutant SOD1 induces aggregation through trimeric SOD1 formation

d

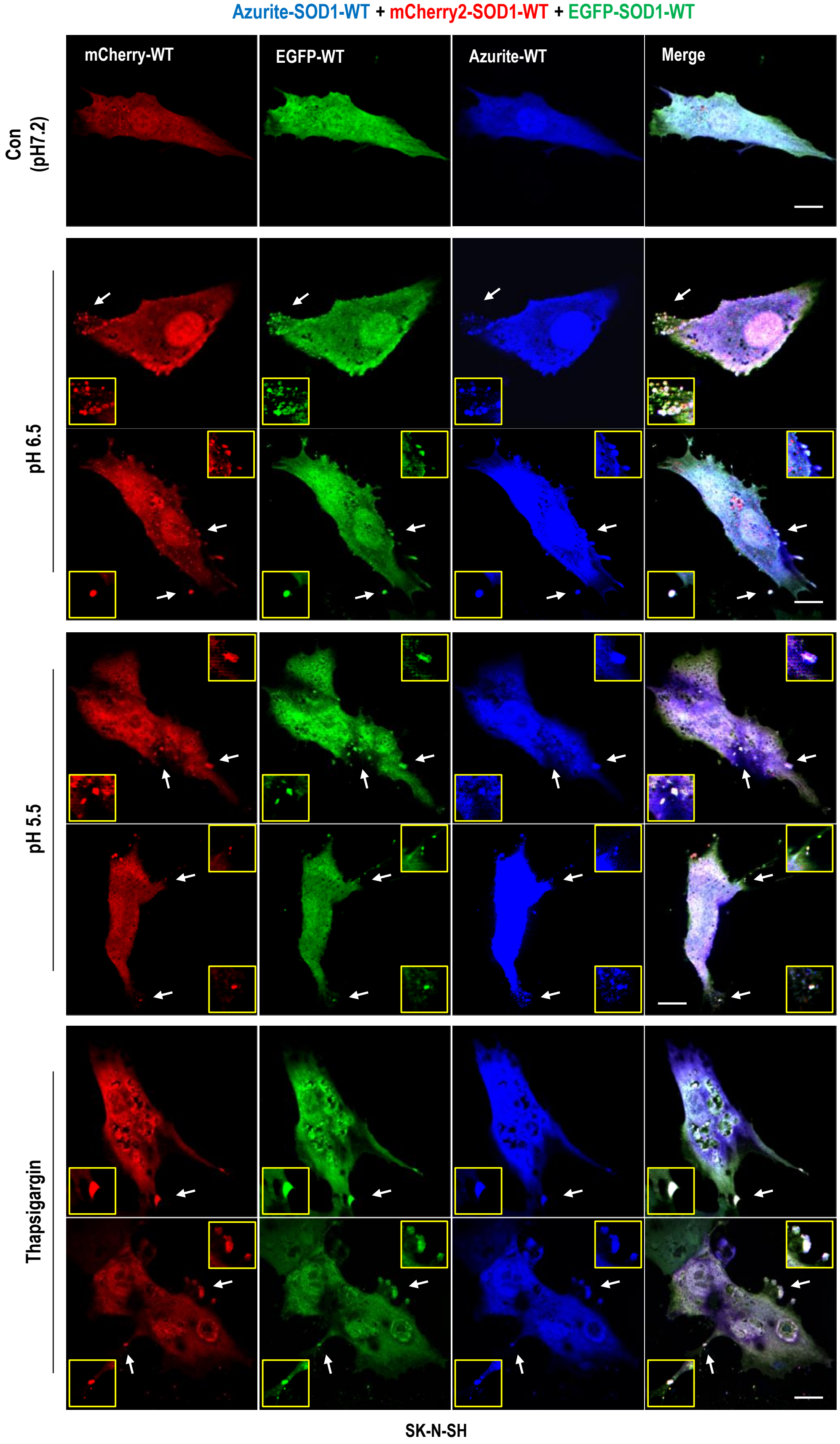

E

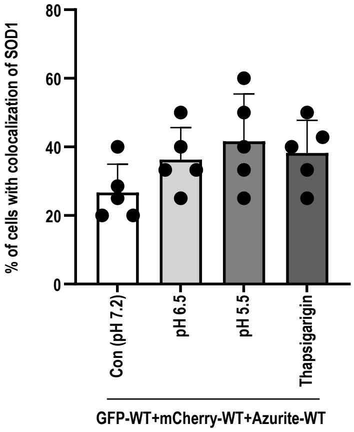

Fig. S2 Mutant SOD1 induces aggregation through trimeric SOD1 formation. a, SOD1 oligomerization was abolished by trimer-destabilizing mutant of SOD1. HEK293 cells were co-transfected with pathological MT-SOD1 (G85R, G93A) expression vectors and SOD1 trimer-destabilizing mutant (N53I, D101I) expression vectors. After incubation, transfected cells were harvested with TNNI buffer and separated using SDS-PAGE. EV is Empty Vector. **b,** Calibration curve for the LC-MS/MS analysis to calculate of binding affinity of PRG-A-04 to GST-SOD1. Diagram for ELISA-based chemical screening system. **c,** PRG-A-04 abolished three different color variants of SOD1 co-inclusions by MT and WT-SOD1 overexpression. Cells were transfected with WT-SOD1 (Azurite and mCherry2) and G85R-SOD1 (GFP) vectors for 24 hr. After incubation, cells were treated with PRG-A-04 (5 μM) for 24 hr and fixed with PFA. The fluorescence were detected for monitoring SOD1 inclusion under Apotome microscope (white arrows; strong intensity and co-localization of SOD1) Scale bar; 10 μm. **d-e,** Three types of WT-SOD1 were co-localized by exposed to cellular stresses in SK-N-SH cells. (d) Cells were transfected with WT-SOD1 (Azurite, mCherry2 and GFP) vectors for 24 hr. After incubation, cells were treated with cellular stresses (low pH and Thapsigargin; Ca^{2+} chelators) for 24 hr and fixed with PFA. SOD1 expression was observed under Apotome microscope (white arrows; strong intensity and co-localization of SOD1) Scale bar; 10 μm. (e) For cell counting, five fluorescence images using Apotome microscope were randomly selected. Counting of cells were performed by three independent observers, who were blinded to transfection or chemical treatment group

Fig. S3. SOD1 protein uptake into the cells through endocytosis

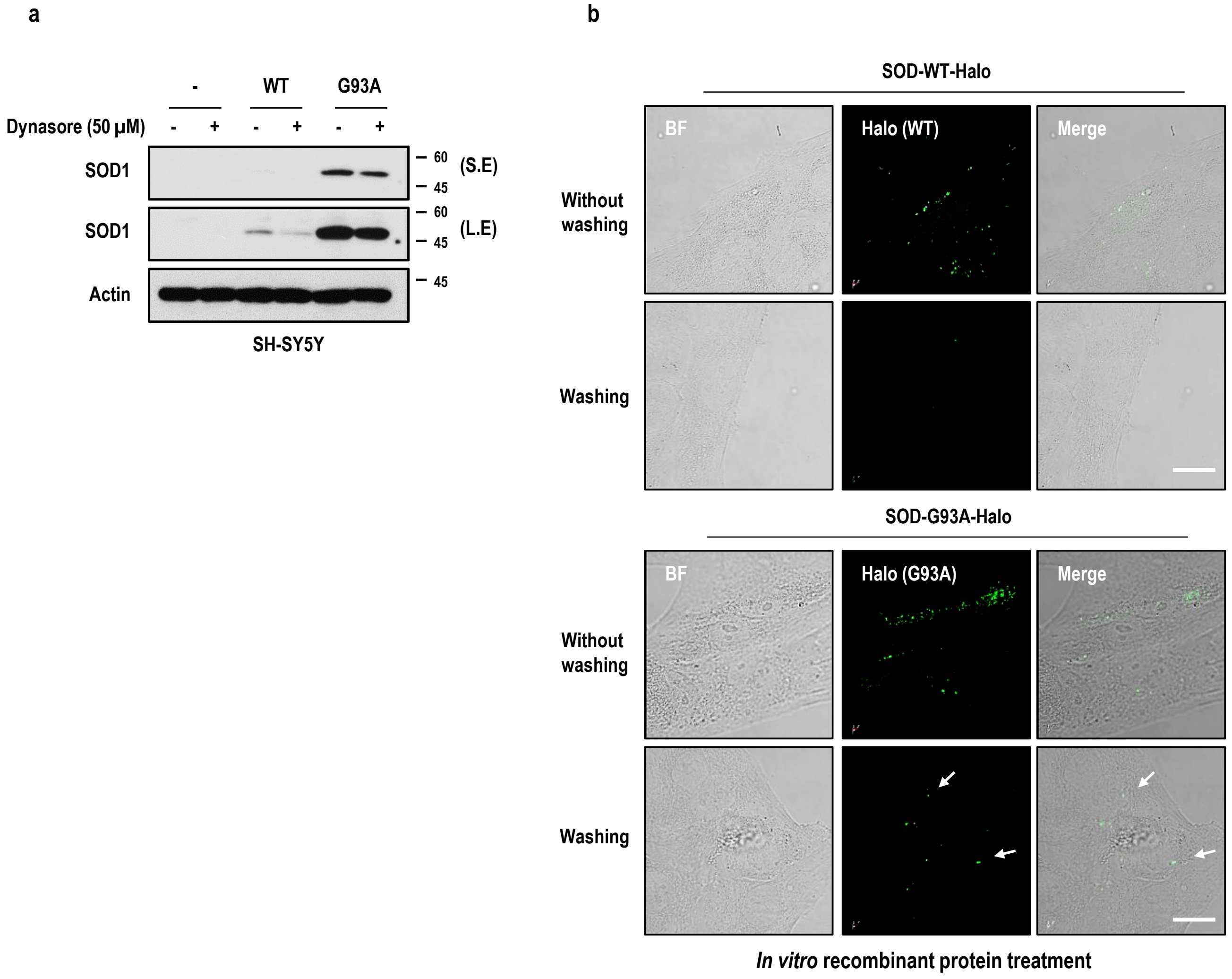

Fig. S3 SOD1 protein uptake into the cells through endocytosis. a, The uptake of SOD1 proteins was achieved by endocytosis. SH-SY5Y cells were treated with SOD1 recombinant proteins and endocytosis inhibitor (Dynasore; 50 μM) for 6hr. After incubations, cells were harvested with RIPA and separated by SDS-PAGE. S.E: short exposure, L.E: long exposure. **b,** The recombinant MT-SOD1 protein is located intracellular space in neuron cells. SOD1-Halo proteins (3 μg/ml) were treated in neuron cells for 2 hr and incubated with HaloTag-fluorscent ligands (cell permeable ligands ; diAcFAM) for 30 min. After incubation, cells were washed with fresh culture medium three times and. Incubate cells in complete culture medium at CO_2 incubator for 30 min to wash unbound ligand. The fluorescence images of live cells were obtained using a commercial holotomography (HT-2H). White arrows indicate the intensity of SOD1. BF; Bright field, Scale bar; 400 μm.

Fig. S4.Therapeutic effect of PRG-A-04 in SOD1$^{G93A-Tg}$ mouse model

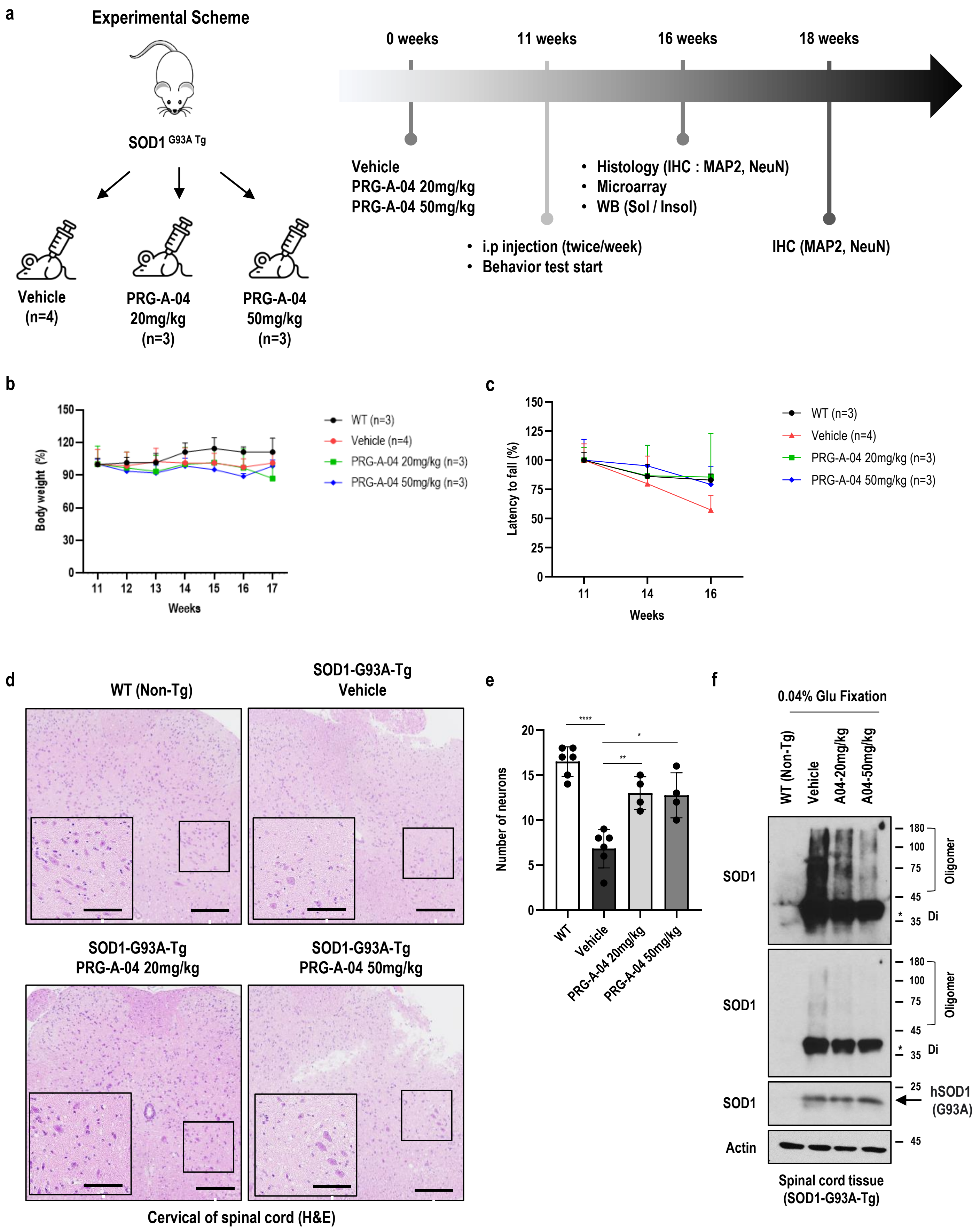

Fig. S4.Therapeutic effect of PRG-A-04 in SOD1$^{G93A-Tg}$ mouse model

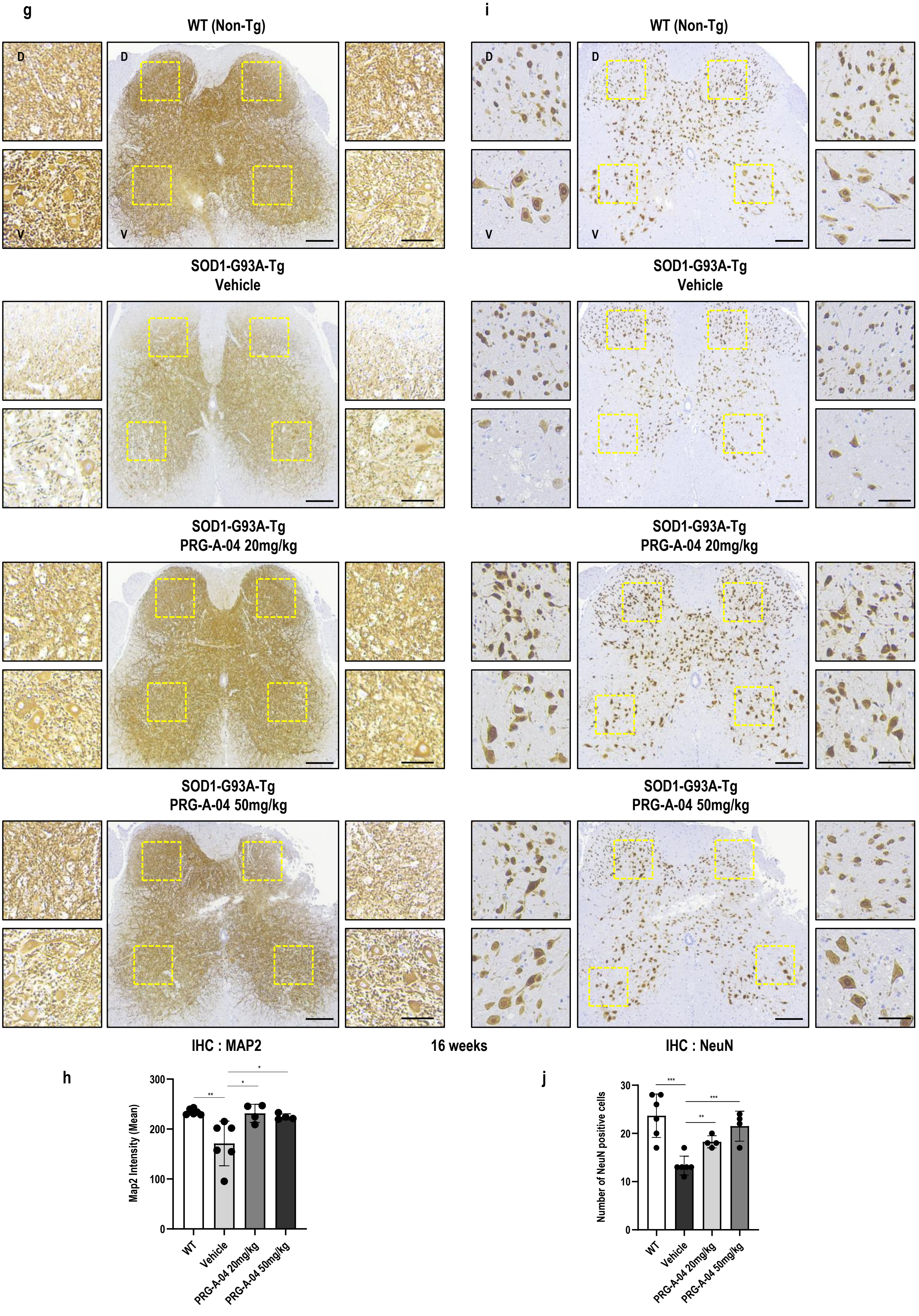

Fig. S4 Therapeutic effect of PRG-A-04 through i.p injection on the SOD1$^{G93A-Tg}$ mouse. a, Experimental scheme for monitoring therapeutic effect of PRG-A-04 in the SOD1$^{G93A-Tg}$ ALS mouse model. 11 weeks old mice were injected with 20 mg/kg and 50 mg/kg, twice per week for 6~8 weeks and checked the motility with rotarod test. To check the histology analysis, some mouse groups were sacrificed and the spinal cord was isolated at 16 weeks and 18 weeks. **b,** PRG-A-04 showed no cytotoxicity in body weight loss. **c,** PRG-A-04 treated groups were maintained the latency to fall with rotarod test. **d-e**, The number of neurons in spinal cord was protected in PRG-A-04 treated mouse groups (n=4), compared to vehicle-treated mice (n=4). (d) The 11 weeks old mice were injected with PRG-A-04 for 6 weeks and sacrificed at 16 weeks. The cervical spinal cord was stained with H&E. Scale bar; 20 μm. (e) Representative images were showed and number of neurons were counted and plotted in right panel. $^{**}P < 0.01$, $^{***}P < 0.005$. **f,** The SOD1 oligomerization was reduced with PRG-A-04 in the cervical spinal cord tissue lysates of mice carrying SOD1-G93A mutation. The tissue lysates were harvest with TNNI buffer and incubation for 30 min then fixed with 0.04% glutaraldehyde (Glu) for 30min. Samples were separated using SDS-PAGE. Arrow indicates human SOD1(hSOD1-G93A) was indicated. **g-j,** PRG-A-04 ameliorated the reduction of MAP2 (g-h) and NeuN (i-j) in the cervical spinal cord of SOD1$^{G93A-Tg}$ mice. The neuronal markers were maintained in dorsal and ventral horn. Scale bar; 20 μm. Representative images were showed with a magnification of 10x and an insert of 20x (yellow box). The intensity of MAP2 (e) and the number of NeuN positive cells (f) were counted with Image J software.

Fig. S5. PRG-A-04 maintains the movement disorder on the SOD1$^{G93A\text{-}Tg}$ mouse model

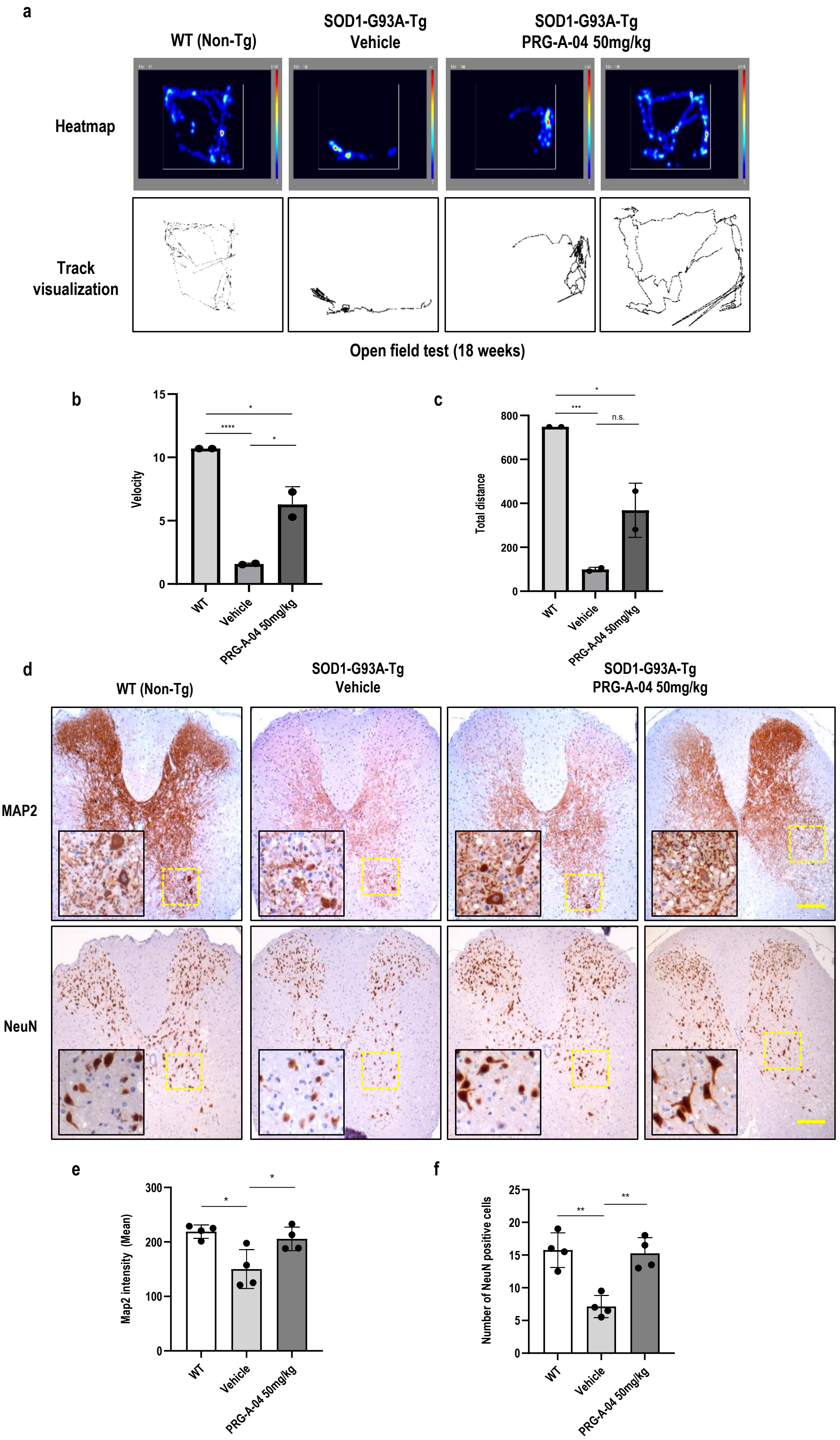

Fig. S5 PRG-A-04 maintains movement disorder on the $SOD1^{G93A-Tg}$ mouse model. a, Open field test of $SOD1^{G93A-Tg}$ mice with PRG-A-04 showed favorable therapeutic effect on motility. Vehicle (DMSO, n=2) and PRG-A-04 treated mice (50 mg/kg, n=2) movement were monitored. Age-matched WT mice (n=2) were tested as positive control. Track visualization analysis was conducted by open field test and heatmap was shown. **b-c,** Velocity (b) and total distance (c) of mice carrying SOD1-G93A mutation was improved by PRG-A-04 treaetment. **d-f,** The cervical of spinal cord was stained with MAP2 and NeuN. The neuronal markers were maintained in dorsal and ventral horn. Scale bar; 20 μm. (d) Representative images were showed with a magnification of 10x and an insert of 20x (yellow box). The intensity of MAP2 (e) and the number of NeuN positive cells (f) were counted. *$P < 0.05$, **$P < 0.001$, ***$P < 0.005$.

Fig. S6. PRG-A-04 improves muscle contraction-related gene expression

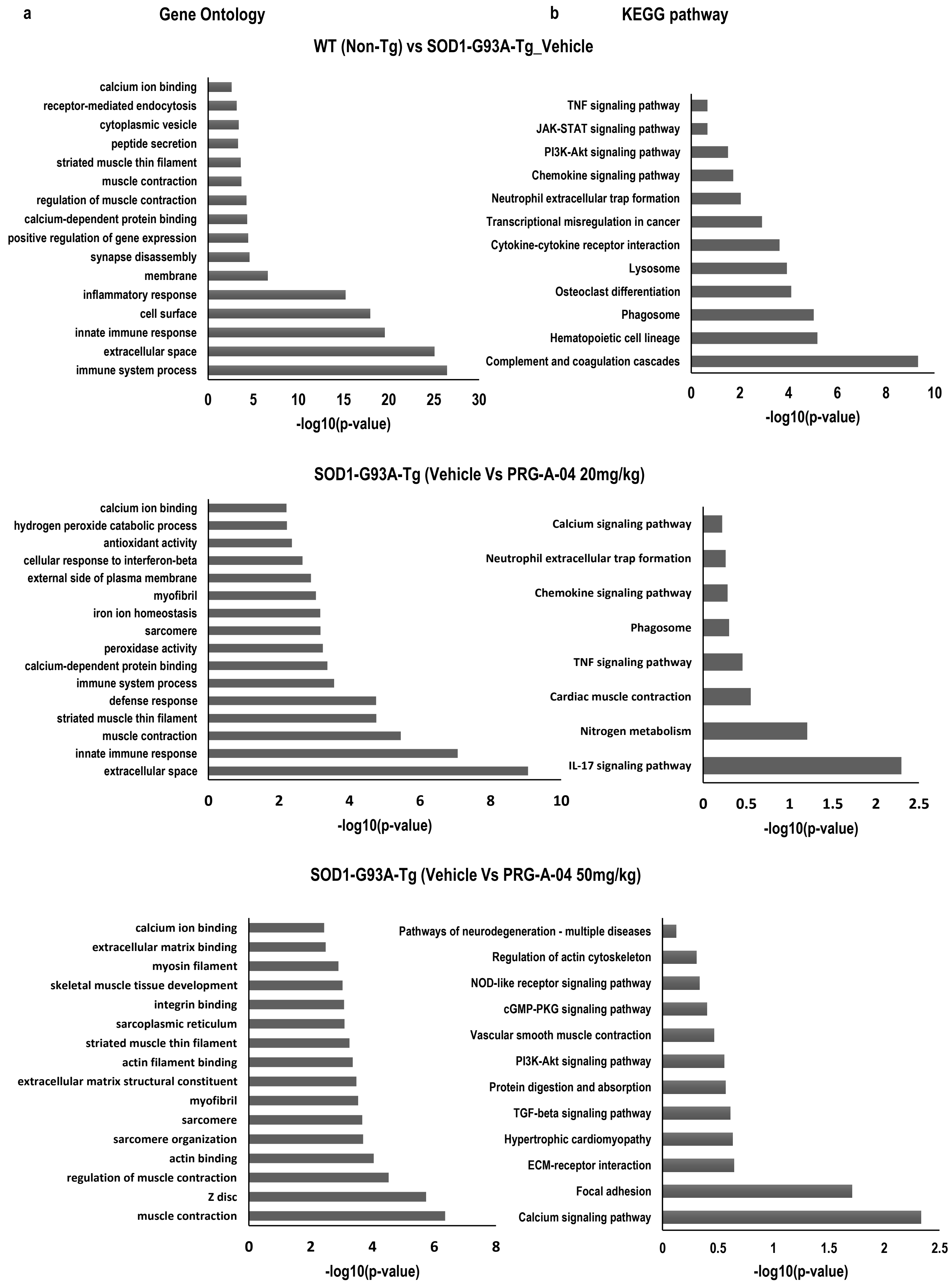

Fig. S6 PRG-A-04 improves muscle contraction related gene expression. a-b. GO term analysis (a) and KEGG pathway analysis (b) of differentially expressed genes between the vehicle and PRG-A-04 treated $SOD1^{G93A\text{-}Tg}$ mice. GO term indicated that PRG-A-04 regulated motility associated gene sets, including muscle contraction, actin binding, sarcomere, Ca^{2+} ion binding. KEGG pathway suggested that PRG-A-04 controlled the pathway including Ca^{2+} signaling, protein digestion and actin cytoskeleton.

Fig. S7. PRG-A-04 extended the life-span via oral administration

a Scheme of in vivo Feeding + i.p efficacy test

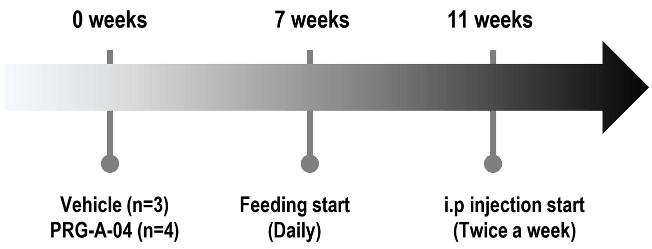

b Composition of pre-mixed drug-diet pellet

Dose	PRG-A-04 (mg)	Solvent (ul) PVP 2%+SLS 1%	Diet (g)
Vehicle	0	2.5ul	2.5
PRG-A-04 : 40mg/kg (0.8mg/20g/day)	0.8	2.5ul	2.5

c

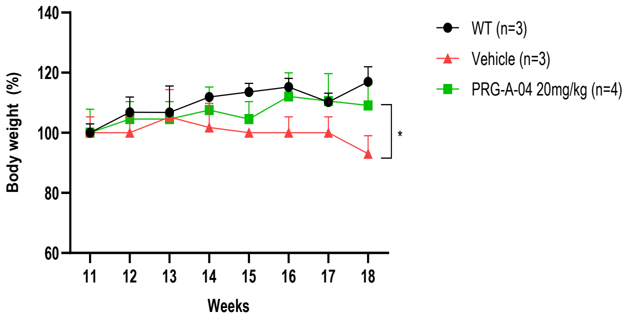

e

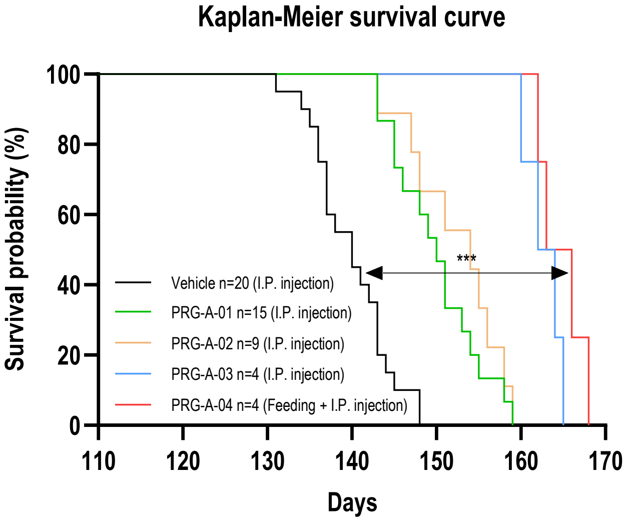

d

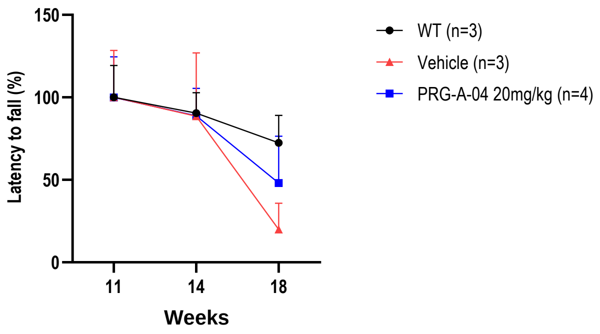

f Experimental Scheme (Start at 9 weeks)

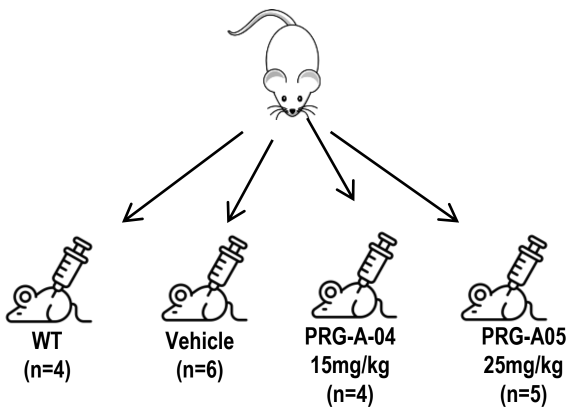

Scheme of in vivo P.O efficacy test (PRG-A-04; ASD)

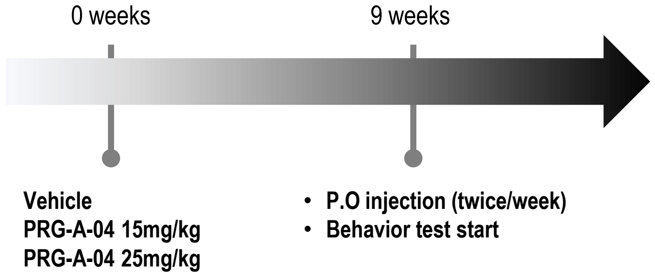

g

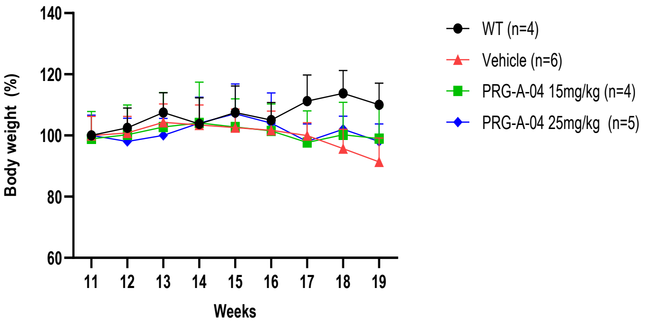

h

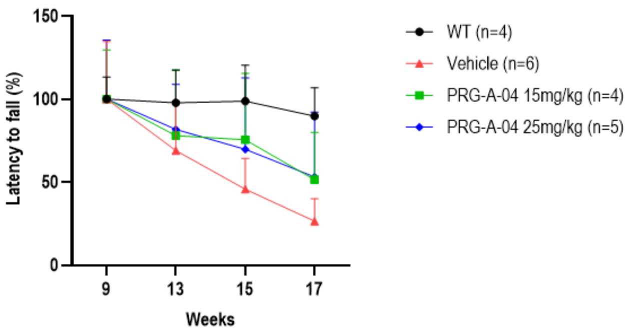

Fig. S7 PRG-A-04 extended the lifespan of SOD1$^{G93A-Tg}$ mice through oral administration. a, Experimental scheme to monitor the combined therapeutic effect of feeding and i.p injection of PRG-A-04 in the SOD1$^{G93A-Tg}$ ALS mouse model. After grouping into vehicle (n=3) or PRG-A-04 (n=4) treated mice, they were fed daily with pre-mixed drug-diet pellets starting at 7 weeks of age, and twice-weekly i.p injections were started at 11 weeks of age to determine longevity. **b**, Compositions of pre-mixed drug diet pellet **c-d**, Comparing to vehicle treated mice, the combination therapy treated mice were maintained the body weight and protected the movement disorder. **e**, Kaplan Meier survival curve of SOD1$^{G93A-Tg}$ ALS mouse model. Comparing to vehicle treated mice, PRG-A-04 treated mice by combined treatment showed extended life span about 25 days. Also, the combination treatment with PRG-A-04 showed an improved life extending effect compared to other derivatives, PRG-A-series. In case of PRG-A-01,02 and 03 were treated with i.p injection for 20 mg/kg on the SOD1$^{G93A-Tg}$. Lifespan of PRG-A-01 data was collected from previous our papers. **f,** Experimental design to monitor the therapeutic effect of PO injection of PRG-A-04 in the SOD1$^{G93A-Tg}$ ALS mouse model. After grouping into vehicle (n=6) and PRG-A-04 [15 mg/kg, (n=4), 25 mg/kg, (n=5)], they were treated with twice-weekly PO injections were started at 9 weeks of age. **g-h**, Comparing to vehicle treated mice, the PRG-A-04 orally administered mice group were maintained the body weight and protected the movement disorder. Extended lifespan data was shown at Fig 4g. $***P < 0.005$.

Fig. S8. Preclinical data of PRG-A-04

a Plasma protein binding

Species/Strain	Concentration (uM)	% Bound
Mouse	0.2	99.91
	2	99.92
	10	99.87
Rat	0.2	99.9
	2	99.91
	10	99.86
Dog	0.2	>99.93
	2	99.95
	10	99.92
Monkey	0.2	>99.94
	2	99.93
	10	99.89
Human	0.2	>99.92
	2	99.97
	10	99.97

b CYP inhibition(direct)

CYP	IC50(μM), DI
1A2	>10.0
2B6	>10.0
2C8	>10.0
2C9	>10.0
**2C19**	**4.75**
2D6	>10.0
3A(midazolam)	>10.0
3A(testosterone)	>10.0

c BBB-PAMPA

Compound	Test Concentration (μM)	Incubation Time (hour)	Mean Pe (nm/s)	Mean % Solution Recovery	Permeability
Atenolol	10	4	<0.184	<109.3	Low
Propranolol	10	4	76.6	82.4	High
PRG-A-04	10	4	4.18	10.4	Moderate

d Mouse Brain Tissue penetration

Plasma Concentration (ng/mL)						
Time (h)	M01+3n	M02+3n	M03+3n	Mean		SD
**0.500**	1370	1870	1930	1723	±	307
**1.00**	1700	1830	1720	1750	±	70.0
**12.0**	1010	881	764	885	±	123

Brain Concentration (ng/g)						
Time (h)	M01+3n	M02+3n	M03+3n	Mean		SD
**0.500**	2980	2880	3660	3173	±	424
**1.00**	3890	3690	3470	3683	±	210
**12.0**	1940	1650	1310	1633	±	315

Brain/Plasma Ratio							
Time (h)	M01+3n	M02+3n	M03+3n	Mean		SD	CV (%)
**0.500**	2.18	1.54	1.90	1.87	±	0.318	17.0
**1.00**	2.29	2.02	2.02	2.11	±	0.157	7.43
**12.0**	1.92	1.87	1.71	1.84	±	0.108	5.88

Fig. S8 Preclinical data of PRG-A-04. a, The plasma protein binding data indicated that PRG-A-04 exhibited a very high binding to plasma proteins in the five species, at all three test concentrations (0.2, 2 and 10 μM). No significant differences in protein binding were observed across species. **b**, PRG-A-04 inhibited CYP2C19 with the IC50 value of 4.75 μM. **c**, BBB-PAMPA assay showed that PRG-A-04 (10 μM) exhibited the moderate permeability The signal responses of atenolol in receiver samples were undetectable (low permeability). For the convenience of calculation, 1/300 of the measured peak area ratio (PAR) of the relevant AD samples were used as the PAR values of atenolol in receiver samples. **d**, Mouse brain tissue penetration assay showed that PRG-A-04 (20 mg/kg) passed through the BBB with a brain/plasma ratio of 2.11
