# Supplementary material for: Inhibition of SOD1 trimerization is a novel drug target for ALS disease
Source: Transl Neurodegener. 2025 May 12;14:21. doi: 10.1186/s40035-025-00483-8 (PMC12067741; doi:10.1186/s40035-025-00483-8)
Supplement: Supplementary file 6 — Additional file 6. Raw data. [file 40035_2025_483_MOESM6_ESM.pptx]

## Slide 1
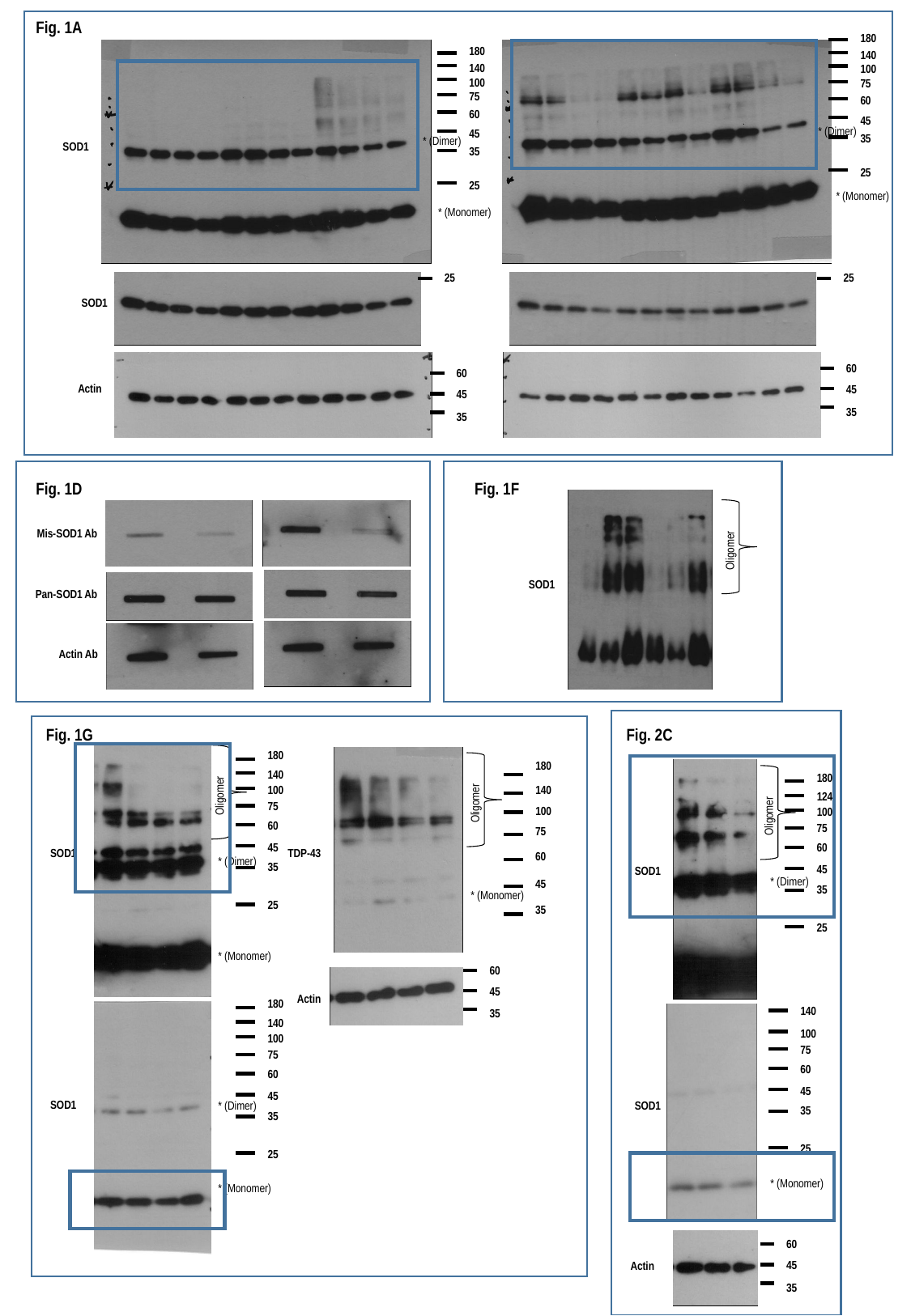

Fig. 1A
180
140
100
75
60
45
35
25
180
140
100
75
60
45
35
25
* (Dimer)
* (Dimer)
SOD1
* (Monomer)
* (Monomer)
25
25
SOD1
60
45
35
60
45
35
Actin
Fig. 1D
Fig. 1F
Mis-SOD1 Ab
Oligomer
SOD1
Pan-SOD1 Ab
Actin Ab
Fig. 1G
Fig. 2C
180
140
100
75
60
45
35
25
180
140
100
75
60
45
35
180
124
100
75
60
45
35
25
Oligomer
Oligomer
Oligomer
SOD1
TDP-43
* (Dimer)
SOD1
* (Dimer)
* (Monomer)
* (Monomer)
60
45
35
Actin
180
140
100
75
60
45
35
25
140
100
75
60
45
35
25
SOD1
* (Dimer)
SOD1
* (Monomer)
* (Monomer)
60
45
35
Actin

## Slide 2
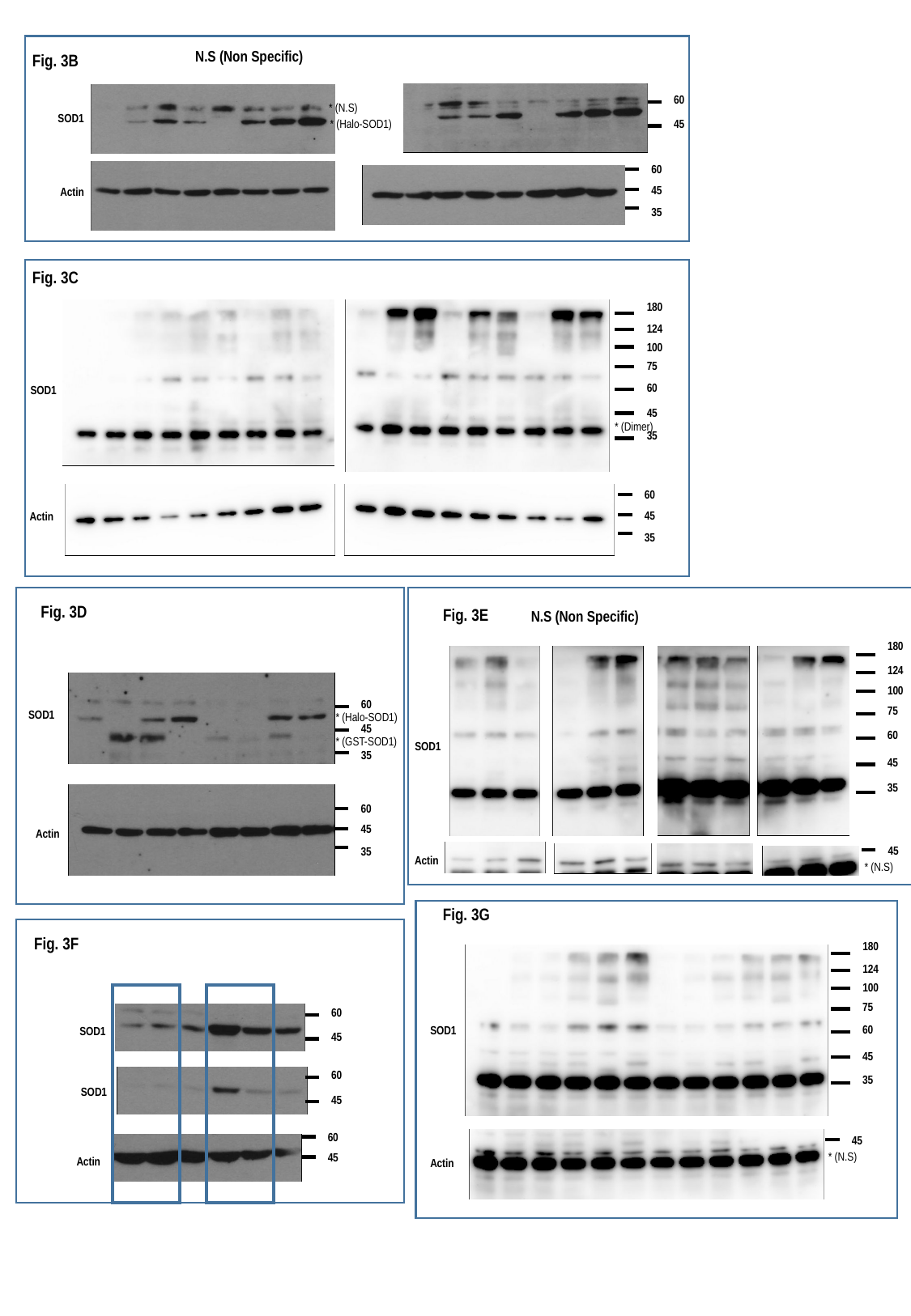

N.S (Non Specific)
Fig. 3B
60
45
* (N.S)
SOD1
* (Halo-SOD1)
60
45
35
Actin
Fig. 3C
180
124
100
75
60
45
35
SOD1
* (Dimer)
60
45
35
Actin
Fig. 3D
Fig. 3E
N.S (Non Specific)
180
124
100
75
60
45
35
60
45
35
SOD1
* (Halo-SOD1)
* (GST-SOD1)
SOD1
60
45
35
Actin
45
Actin
* (N.S)
Fig. 3G
Fig. 3F
180
124
100
75
60
45
35
60
45
SOD1
SOD1
60
45
SOD1
60
45
45
* (N.S)
Actin
Actin

## Slide 3
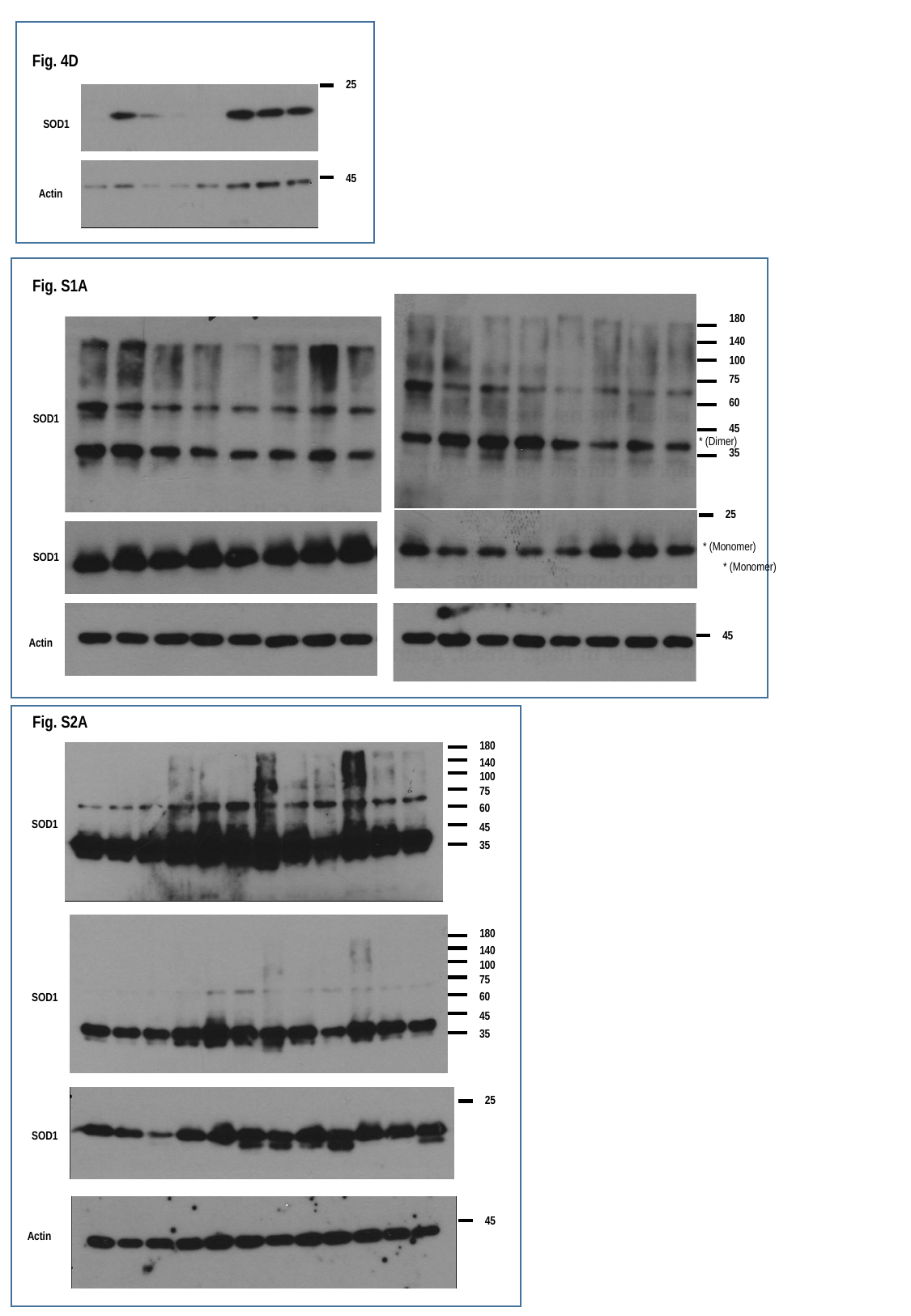

Fig. 4D
25
SOD1
45
Actin
Fig. S1A
180
140
100
75
60
45
35
SOD1
* (Dimer)
25
* (Monomer)
SOD1
* (Monomer)
45
Actin
Fig. S2A
180
140
100
75
60
45
35
SOD1
180
140
100
75
60
45
35
SOD1
25
SOD1
45
Actin

## Slide 4
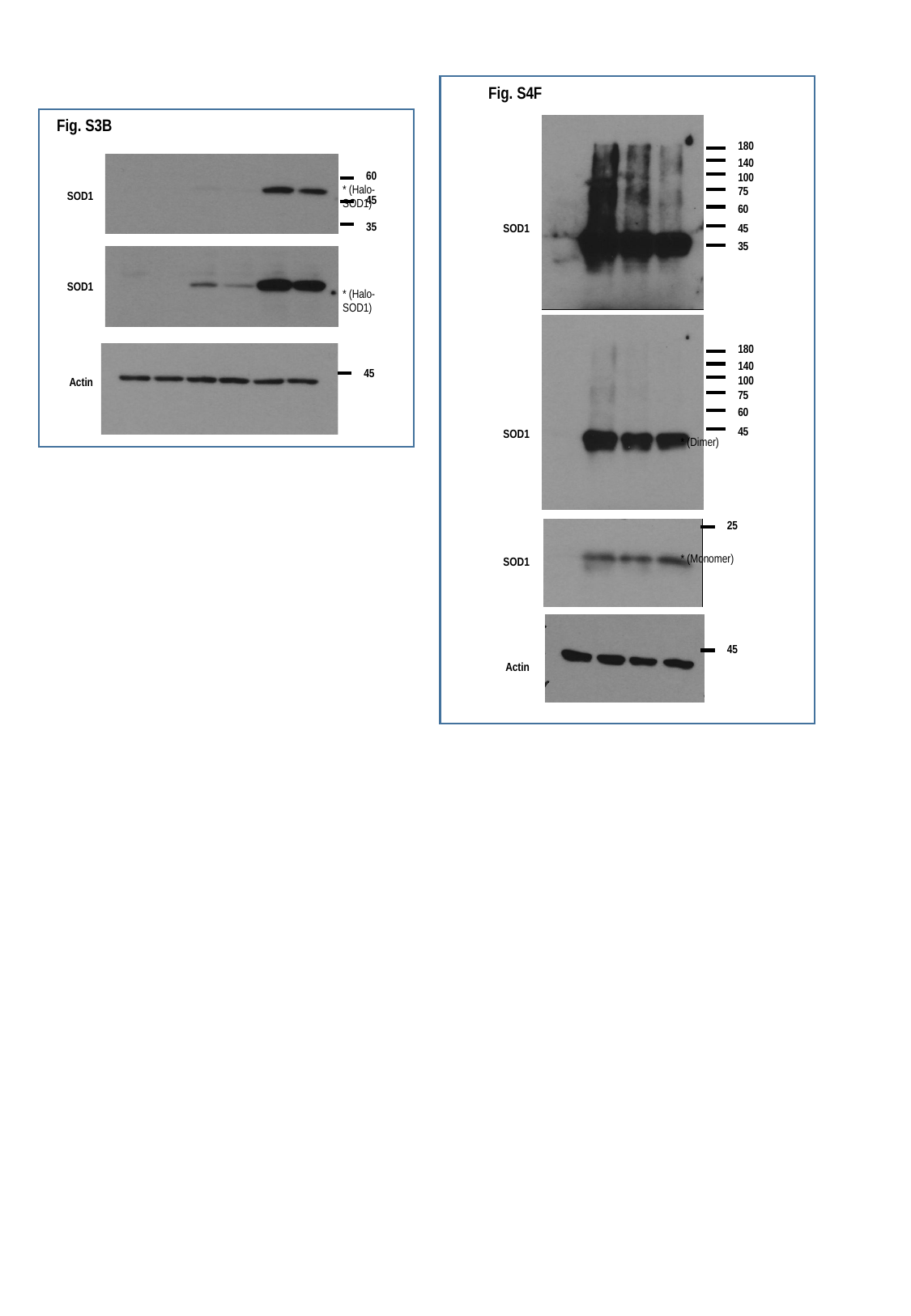

Fig. S4F
Fig. S3B
180
140
100
75
60
45
35
60
45
35
* (Halo-SOD1)
SOD1
SOD1
SOD1
* (Halo-SOD1)
180
140
100
75
60
45
45
Actin
SOD1
* (Dimer)
25
* (Monomer)
SOD1
45
Actin
